# Diagnosis of Indian Big Four and monocled Cobra snakebites in envenomed plasma using smartphone-based digital imaging colourimetry method

**DOI:** 10.1371/journal.pntd.0012913

**Published:** 2025-03-14

**Authors:** Upasana Puzari, Mojibur R. Khan, Ashis K. Mukherjee

**Affiliations:** 1 Microbial Biotechnology and Protein Research Laboratory, Department of Molecular Biology and Biotechnology, School of Sciences, Tezpur University, Tezpur, Assam, India; 2 Division of Life Sciences, Institute of Advanced Study in Science and Technology, Vigyan Path Garchuk, Paschim Boragaon, Guwahati, Assam, India; 3 Academy of Science and Innovative Research (AcSIR), Ghaziabad, India; Fundação de Medicina Tropical Doutor Heitor Vieira Dourado: Fundacao de Medicina Tropical Doutor Heitor Vieira Dourado, BRAZIL

## Abstract

**Background:**

Venomous or dry bites can result from snake envenomation. Therefore, developing a detection test for venomous snakebites in envenomed patients can prevent from unnecessary antivenom therapy for dry bites, thereby, saving them from adverse effects and cost of antivenom therapy.

**Methodology:**

This study demonstrates a method for the diagnosis of medically significant ‘Big Four’ Indian snake venoms (*Naja naja, Bungarus caeruleus, Daboia russelii, Echis carinatus*) in the plasma of experimentally envenomed animals (envenomed under laboratory conditions). Rabbit polyclonal antibodies (PAbs) were produced by generating modified bespoke peptides identified by computational analysis from the antigenic sites of the main toxins found in the proteome of India’s ‘Big Four’ venomous snakes. The polyclonal antibody formulation (FPAb) prepared by mixing the five representative PAbs in the ratio of 1:1:1:1:1 demonstrated synergistic immune recognition of the ‘Big Four’ snakes and *Naja kaouthia* venoms. The recognition for these venoms under *in vitro* and *in vivo* conditions by FPAb was significantly higher (p<0.05) than commercial polyvalent antivenom produced against native venom toxins. The FPAb was tested to detect the venoms in subcutaneously envenomed rat plasmas until 240 minutes post-injection. Fourier-transform infrared spectroscopy, zeta potential, transmission electron microscopy, and atomic force microscopy characterised gold nanoparticles (AuNP) conjugated with FPAb. The FPAb-conjugated AuNP demonstrated aggregation upon interaction with venom toxins, changing the colour from red through burgundy to blue, monitored using a smartphone. From the digital image colourimetry analysis of the images, calibration curves for venoms were obtained, and each venom in the envenomed plasma at different time intervals was quantified using these curves.

**Conclusion:**

A method for detection of venomous snakebites has been reported. The formulation of polyclonal antibodies generated against toxins of ‘Big Four’ venomous snakes of India immune-recognise venoms of ‘Big Four’ venomous snakes of India and *N. kaouthia* venoms under both *in vitro* and *in vivo* conditions. The antibody formulation conjugated to AuNP detected the venoms in envenomed plasma. This method of detection has potential to be useful for snakebite management in clinical settings.

## Author summary

There is an urgent need to develop a detection test for venomous snakebites in patients. In this study we have reported a method for diagnosis of venoms from the ‘Big Four’ Indian snakes and *Naja kaouthia* in the plasma of Wistar strain albino rats injected with these venoms. Custom peptides designed and synthesized from toxins identified from the proteome of ‘Big Four’ venomous snakes of India were used to raise five polyclonal antibodies in rabbit. The polyclonal antibodies were combined in a certain ratio to obtain a formulation. The antibody formulation demonstrated recognition for the ‘Big Four’ snakes and *Naja kaouthia* venoms under *in vitro* and *in vivo* conditions and these recognitions were higher than that of commercial polyvalent antivenom. Wistar rats were subcutaneously injected with these venoms and the antibody formulation was able to detect the venoms in envenomed rat plasma until 4 hours post-injection. The antibody formulation was further conjugated to gold nanoparticles and the conjugate was characterised by biophysical techniques such as Fourier-transform infrared spectroscopy, zeta potential, transmission electron microscope and atomic force microscopy. Upon interaction with the envenomed plasma, the antibody formulation conjugated to gold nanoparticles changing colour from burgundy to blue which was recorded using smartphone camera. Calibration curves for the venoms were attained after colourimetry analysis of smartphone images and these curves were used to quantify the venom in envenomed plasma at different time intervals.

## Introduction

Snake envenomation, a neglected public health issue, frequently results in life-threatening situations in tropical and subtropical nations. An estimated 5.4 million snake bite incidents are reported annually, translating into 1.8 - 2.7 million instances of envenomation [1]. Asia alone reports about 2 million bites from venomous snakes yearly [2]. The Registrar General of India-Million Death Study (RGI-MDS) has estimated that there are around 46,900 deaths per year due to venomous snakebites in India [2]. The magnitude of snake envenomation issue is far greater than documented in the published material.

The Indian peninsula hosts over 52 deadly species of snakes. The Indian cobra (*Naja naja*), Indian common krait (*Bungarus caeruleus*), Indian Russell’s viper (*Daboia russelii russelii*), and Indian saw-scaled viper (*Echis carinatus*) are collectively referred to as the ‘Big Four’ venomous snakes of India, as they account for the predominant incidence of morbidity and death in these areas. [3–5]. India’s ‘Big Four’ are categorised as category I medically important snakes, necessitating rapid medical intervention upon envenomation. In the northeastern region of India, different snake species, such as the Indian monocled cobra (*N. kaouthia*) and many kinds of pit vipers, contribute to numerous fatalities [5–7].

Snakebite from a venomous snake may be either a ‘wet bite’ (venom injection) resulting in mild local symptoms to severe systemic toxicity and ultimately death, or they may be a ‘dry bite’ without local or systemic signs of envenomation [4,6,8]. Potential causes of ‘dry bite’ in various snake species may include a small amount of venom injection or no venom injection owing to the shape of the fangs and imprecise venom delivery [8]. Antivenom administration is the only current therapy for snakebite envenomation. However, this therapy has several adverse and costly effects [9]. Hence, using a method or kit to precisely determine whether a particular snakebite case is classified as a ‘wet bite’ or a ‘dry bite’ will significantly deter hospital authorities or physicians from delivering antivenom at every instance of snakebite [8]. The traditional methods of snake venom detection relied on visual confirmation by patients or witnesses, analysis of local symptoms of envenomation, and biochemical analyses of urine and blood [10,11]. Advancements in modern analytical techniques have led to the development of ELISA-based methods, immunofluorescence assays, optical immuno assays, dot-ELISA, enzyme-linked aptamer assays and lateral flow assays for diagnosis of specific venoms and these methods used purified venom-specific antibodies and nucleic acid aptamers for the detection studies [12–18]. Researchers have also utilized polymerase chain reaction (PCR) for the identification of snake species using trace DNA extracted from venom at bite locations [19,20].Despite a range of sensitive and specific detection methods for snake venoms, there have been technical impediments like sophisticated instruments, longer detection time for some methods, cross-reactivity toward several snake species and lack of studies to detect trace quantities of venom post envenomation, which have hindered the commercialization of these methods as point-of-care devices in rural health centres [6].

Snake venom is rich in protein and polypeptide toxins, and some of these toxins are present in the venom in higher quantities. Therefore, antibodies against these toxins may be developed for an efficient snake venom diagnosis kit to detect snake venom in animals’ plasma. However, purifying toxins from snake venom is a laborious and costly affair. An alternative approach may be mapping the antigenic epitopes of these major toxins, synthesising custom peptides, and developing antibodies against these peptides to detect snake venom. Proteomic analyses of venom proteomes of ‘Big Four’ venomous snakes of India from our lab have identified the relative abundances of the toxins [21–28]. Based on the relative abundance of toxins in India’s ‘Big Four’ venomous snakes, we have selected the most abundant venom toxins that exhibit slower absorption into the systemic circulation and slower systemic clearance [29,30].

Digital image colourimetry (DIC) utilizing cell phones has garnered interest among analytical chemists as an increasingly popular analytical technique owing to its simplicity, cost-effectiveness, and mobility [31–33]. Colourimetric assays utilize digital images from various devices such as digital cameras, webcams, scanners, or smartphones, enabling real-time analysis [32,33]. Smartphone cameras begin the image capture process by segmenting the visuals into two-dimensional grids known as pixels. The processing of pixel values through various colour spaces occurs within Android or iOS operating systems, leading to the images that appear on smartphone screens [34]. The CMYK (cyan, magenta, yellow, and black) and RGB (red, green, and blue) are predominant filters. The RGB colour system, designed to replicate human vision, employs three matrices—R, G, and B, to represent the intensity values of these colour components. The intensity values can elucidate the relationships between colour components and analyte concentrations [33,35].

In this study, we have identified the antigenic sequences from the most abundant venom toxins selected from the venom proteome of India’s ‘Big Four’ venomous snakes, by computational (*in silico*) analysis, and modifications were introduced to peptide sequences to enhance their antigenicity for better detection. These modified synthetic peptides were then used to generate high-titre polyclonal antibodies in rabbits (PAbs), and these antibodies were mixed in a particular proportion to obtain an antibodies formulation (FPAb) for better detection of snake venom than the individual antibodies against peptide antigens. Since the sequences of *N. naja* and *N. kaouthia* PLA_2_ have been listed as having 90% similarity in the UniProt database (https://www.uniprot.org/uniprotkb), the immune reactivity of FPAb was tested against ‘Big Four’ venomous snakes of India, as well as against *N. kaouthia* under *in vitro* and *in vivo* conditions. Upon conjugating FPAb to gold nanoparticle (AuNP), we utilised the colourimetric sensing ability of AuNPs where the AuNP-FPAb conjugates changed colour from red to blue/purple on interaction with the snake venoms studied due to aggregation of the particles. This study used high-resolution images provided by smartphone cameras to detect the colour changes, and digital image analysis was performed using an application to determine the condition of the venomous or wet bite. Our study’s antibody formulation may assist in distinguishing between venomous and non-venomous/dry bites in snakebite victims using a smartphone. Thus, this method can potentially be developed as a low-cost and portable method for venom detection at point-of-care.

## Methods

### Ethics statement

All the animal experimentations using the albino Wistar strain rats were carried out after approval from the Institutional Animals Ethics Committee of Institute of Advanced Study in Science and Technology, Guwahati, Assam, India vide approval number, IASST/IAEC/2022/09. The white New Zealand rabbits (*Oryctolagus cuniculus*), were maintained and used according to the guidelines approved by Institutional Animals Ethics Committee of BioBharati Pvt. Ltd. and CCSEA(Committee for Control and Supervision of Experiments on Animals) (Registration number of BioBharati Pvt. Ltd. animal house facility: 2309/PO/Rc/S/2024/CCSEA).

### Materials

Lyophilised venoms of *N. naja* (NnV), *B. caeruleus* (KV), *D.russelii* (RvV) and *E. carinatus* (EcV) were procured from Irula Snake Catchers Co-Operative, Tamil Nadu. Indian monocled cobra (*N. kaouthia)* venom (NkV) was collected from Kamrup district of Assam, North-East India (NEI) under the permission from the Assam State Biodiversity Board, Guwahati (ABB/Permission/2021/114). Lyophilised *Mesobuthus tamulus* venom was a gift from Premium Serum and Vaccines Pvt. Ltd. (PSVPL), Pune, India. Lyophilised commercial anti-snake antivenom (PAV) was procured from PSVPL, Pune, India [batch no.: ASVS(I)ly-015]. All other analytical grade chemicals and reagents utilised in the investigation were procured from Sigma Aldrich, USA, and HiMedia, India. The synthesis of toxin-epitope-specific bespoke peptides and the manufacture of antibodies against these peptides in rabbits were contracted to S. Biochem Pvt. Ltd., India, and BioBharati Pvt. Ltd., Kolkata, India, respectively.

### Animals

This study used laboratory inbreed, pathogen-free Wistar strain albino rats of both sexes aged 2-3 months (220 ± 10 g) purchased from M/s Chakrabarty Enterprise, Kolkata. The CCSEA guidelines were followed for the maintenance and use of the Wistar strain albino rats at the animal house facility of Institute of Advanced Study in Science and Technology (Guwahati). Wistar strain albino rats were maintained at 12:12 h light-dark cycle, room temperature of 22 ± 3 °C with a relative humidity of 30-70% and fed with a standard diet of “Amrut” procured from Krishna Valley Agrotech-LLP, Pune, Maharashtra, India, and water *ad libitum*. Further, at BioBharati Pvt. Ltd. antibody production was carried out using white New Zealand rabbits (*Oryctolagus cuniculus*). The rabbits were kept under a 12:12 hour light-dark cycle, with room temperature regulated between 17-21°C and relative humidity maintained at 30-70%.

### Identification and design of antigenic epitopes for the principal toxins of the ‘Big Four’ snake species of India via an *in-silico* methodology

Identifying and designing custom antigenic epitopes was performed per the previously described protocol [36]. Briefly, sequences of the major toxins of ‘Big Four’ venomous snakes of India, namely, PLA_2_ toxin of *N. naja,* PLA_2_ toxin of *D. russelii,* Echicetin of *E. carinatus,* and β-bungarotoxin and Basic PLA_2_ toxin of *B. caeruleus,* were obtained from the National Centre for Biotechnology Information (NCBI) (https://ncbi.nlm.nih.gov) server. The antigenic region for the retrieved sequences was determined by submitting the sequences to Immunomedicine Group: Predicted Antigenic Peptides online server (http://imed.med.ucm.es/Tools/antigenic.pl) [37], which predicted antigenic regions based on the occurrence of amino acid residues in segmental epitopes known experimentally. Ultimately, antigenic epitopes with antigenic propensity > 1 were selected as antigenic regions for raising antibodies [37]. The peptides were designed for this study by combining two antigenic epitopes identified for each toxin sequence to increase the antigenic propensity and raise polyclonal antibodies in rabbits. Protein-protein BLAST (BLASTp) (https://blast.ncbi.nlm.nih.gov/) searches were performed against a nonredundant protein database under default settings using the custom peptides (CPs) sequences to determine the sequence identity of CP sequences with *N. kaouthia* PLA_2_. Further, multiple sequence alignment (Clustal Omega, EMBL-EMI, https://www.ebi.ac.uk/jdispatcher/msa/clustalo) was performed to demonstrate the alignment of the CP sequences and *N. kaouthia* PLA_2_ sequence.

### Polyclonal antibodies raised against the toxin-specific antigenic CPs

The five CPs were conjugated with carrier protein Keyhole Limpet Hemocyanin (KLH), and these conjugated CPs were used to raise the corresponding polyclonal antibodies in rabbits using the method previously described [36,38]. The detailed protocol has been mentioned in S1 Text. PAb 1 was raised against CP 1, PAb 2 against CP 2, PAb 3 against CP 3, PAb 4 against CP 4 and PAb 5 against CP 5.

After purification, the PAbs were combined in a formulation named FPAb [PAb 1+2+3+4+5 (1:1:1:1:1, w/w/w/w/w)] to determine the synergistic increase in immune recognition of the ‘Big Four’ venomous snakes of India and NkV, as compared to individual PAbs, PAb formulation with the Elapidae toxin-specific antibodies PAbE [PAb 1+4+5 (1:1:1, w/w/w)] and PAb formulation with the Viperidae toxin-specific antibodies PAbV [PAb 2+3 (1:1, w/w)] using the dot blot assay described below.

### Immunoblotting and spectrofluorometric analysis to determine the *in vitro* immune cross-reactivity of PAb formulation and commercial PAV towards snake venoms

#### Western blot analysis.

Western blot analysis was performed as per previous protocols to evaluate the immunorecognition of the Indian snake venoms (NnV, NkV, KV, RvV and EcV) against FPAb and commercial PAV [25,39]. Briefly, 80 μg (protein content) of snake venoms were separated in a 12.5% SDS-PAGE under reduced conditions and transferred to a methanol-activated PVDF membrane using Trans-Blot SD semi-dry cell (Bio-Rad). The transfer efficacy of proteins to the PVDF membrane was determined using 0.5% Ponceau-S red. Post-overnight blocking of the membrane with 5% BSA in TBS-T, at 4°C, to prevent non-specific binding of FPAb, the membrane was washed thrice with TBS-T. The membrane was then incubated with FPAb/commercial PAV as primary antibody (1:1000 dilution) at room temperature for 2 h. The membrane was then incubated with HRP-conjugated anti-rabbit/anti-horse IgG antibody at 1:4000 dilution for 1 h at room temperature and washed thrice with TBS-T. The immunoblot was developed using ECL substrate (Cat no. 1705060, Bio-Rad) using the ChemiDoc imaging system with Image Lab software (Bio-Rad, USA). The aggregate band intensities were computed utilizing ImageQuant analysis software (Cytiva, USA). The lanes in the blots were selected and treated with Subtract Background command to diminish background noise, employing a rolling ball radius of 50 pixels. The bands were detected in the lanes, and quantified.

#### Dot blot analysis.

Dot blot analysis was performed per our previous protocol with slight modifications [36,38]. Briefly, PVDF membranes were activated with 100% methanol for 2 min and washed with TBS-T for 15-30 min. The activated membranes were spotted with 2 μg FPAb/commercial PAV, and non-specific binding was blocked with 5% BSA in TBS-T solution for 1 h. The membranes were incubated with snake venom-spiked rat plasma (1 pg/μL) and *Mesobuthus tamulus* venom-spiked rat plasma (0.3 ng/μL) for 30 min at room temperature and washed thrice with TBS-T. Post-washing, the membranes were incubated for 45 min with FPAb/commercial PAV as the primary antibody at 1:1000 dilution. The HRP-conjugated anti-rabbit/anti-horse IgG detected the primary antibody at 1:2000 dilution.

The immunoblots were developed using the ECL substrate described above. The dot intensities were analysed using ImageJ software (National Institute of Health USA, http://imagej.nih.gov/ij). The Process/Subtract Background command was used to adjust the pictures’ backgrounds to perform dot blot intensity analysis in ImageJ, and the rolling ball radius was set to 25 pixels. After background correction of the photos, the “Integrated Density” option was activated in the Analyze/Set Measurements command, the circular selection tool was dragged over the dots, and measurements were taken using the Analyze/Measure command. The Edit/Invert command inverted the whole picture of the blot in order to calculate integrated density correctly. Since we have used the same antibody for capture and detection, to dissuade any uncertainty about false recognition, we performed another dot blot assay where we incubated the HRP-conjugated anti-rabbit IgG directly with the capture antibody (control without antigen) [36]. Dot intensities of the immunoblots obtained in the previous dot blot assays were normalised against the dot intensities of the control without antigen. The analyses were performed in triplicates.

### Spectrofluorometric analysis to determine the venom-antivenom interactions

Spectrofluorometric analysis was performed using the methodology described previously [36,38–40]. In brief, a fixed concentration of each snake venom (10 μg/mL) was incubated with different concentrations of FPAb/commercial PAV (10 to 1280 μg/mL). For analysis of the reaction mixture, the excitation wavelength was set at 280 nm, temperature at 25°C, emission slits at 5 nm, and emission spectra were recorded from 300 to 500 nm using the Varioskan LUX Multimode Microplate reader (Thermo Fisher Scientific, Denmark). The fluorescence spectra obtained of only venom/only FPAb or commercial PAV were considered as control, and the relative fluorescence spectra intensity (λ_max_) of snake venom-FPAb/commercial PAV interactions were determined by comparing to the control. Change in relative fluorescence spectra intensity (Δλ_max_) was plotted against the FPAb/commercial PAV concentration (mg/mL) to obtain a graph using GraphPad Prism 5.0 software [41,42], and the K_D_ values were calculated from the graph.

### Determination of *in vivo* immune cross-reactivity of the FPAb and commercial PAV towards snake venoms in the plasma of envenomed animal model

#### Envenomation of animal model.

Albino Wistar strain rats were used to stimulate the experimental envenomation as described previously [36]. The experimental rats were divided into six groups (n=30). Individual groups of albino Wistar strain rats (220 ± 10 g, n=5) were subcutaneously injected with different dosages of each venom. Attention was given to the animal’s minimum pain during the entire experiment. Group 1 rats (n=5) were injected with subcutaneous injections of 1X PBS and served as controls. Further, five groups of rats (groups 2, 3, 4, 5, and 6, n=5) were injected subcutaneously with the respective venoms (NnV, KV, RvV, EcV, and NkV) dissolved in 200 µL of 1X PBS.

The groups of rats (n=5) used herein are as mentioned: - Group 1: Subcutaneous (s.c.) injection of 1X PBS; Group 2: S.c. injection of NnV (0.28 mg/kg); Group 3: s.c. injection of KV (0.16 mg/kg); Group 4: s.c. injection of RvV (0.28 mg/kg); Group 5: s.c. injection of EcV (0.56 mg/kg); Group 6: s.c. injection of NkV (0.56 mg/kg).

### Determination of immune-reactivity of the FPAb and commercial PAV towards Indian snake venoms in the plasma of envenomed animal model

From each animal of the control and envenomed rat groups, blood collection was done from their retro-orbital plexus at the intervals of 60 min, 120 min, and 240 min, post-venom injection, in tubes containing heparin as an anticoagulant (5% of the total volume of blood collected). The blood collected in the tubes was centrifuged to separate the plasma on a fixed rotor centrifuge at 4°C with 4300 rpm for 15 min. The plasma was stored at -20°C and used within seven days [36]. As mentioned in the above section, the plasma samples were analysed using dot blot analysis.

### Gold nanoparticle-based detection of snake venom in the plasma of envenomed animals

#### Synthesis of gold nanoparticles (AuNPs).

Gold nanoparticles were synthesised using the sodium citrate reduction method modified and described in our previous study [36]. Concisely, 1 mM of chloroauric acid solution (HAuCl_4_) (45 mL) was brought up to reflux under stirring conditions. Once the solution started boiling, 38 mM sodium citrate solution (5 mL) was added. When colour of the reaction mixture changed from pale yellow to wine-red, it was cooled to room temperature. The synthesised gold nanoparticle solution was stored at 4°C in an amber bottle.

The AuNPs were then characterised by ultraviolet-visible (UV-Vis) spectrophotometer, Fourier-transform infrared spectroscopy (FTIR), Zetasizer, transmission electron microscope (TEM), and atomic force microscopy (AFM) [36]. The UV-Vis spectra of the synthesized AuNPs were acquired using a Shimadzu UV spectrophotometer UV-2600 within the wavelength range of 400-700 nm. FTIR spectroscopy was conducted to document the chemical bonds produced during the production of AuNPs, utilizing the Nicolet 6700 FTIR equipment with KBr pellets. The Zetasizer Nano ZS, 90 (Malvern, UK) equipment was utilized to assess the surface zeta potential of the synthesized AuNPs. For TEM examination, the synthesised AuNPs were deposited onto a carbon-coated copper grid, and pictures of the citrate-capped AuNPs were captured using a JEOL TEM-2100 plus model and then processed with ImageJ software. Finally, the produced AuNPs were dried on glass slides at ambient temperature and examined using an atomic force microscope (NTEGRA PRIMA, NT-MDT technology) in non-contact mode [43]. The Gwyddion software (Department of Nanometrology, Czech Metrology Institute) was employed to examine the AFM pictures, while the heights of the AuNP particles were computed using the Gaussian function in Origin software.

#### Synthesis of AuNP-FPAb conjugates.

Conjugation of the antibody formulation with AuNPs was carried out by EDC-NHS covalent coupling using our previous protocol [36]. The detailed protocol is mentioned in S1 Text. Bradford’s protein estimation method was used with BSA as a standard to determine the concentration of unbound antibodies remaining in the supernatant after the conjugation. The binding efficiency of the FPAb was calculated using the following equation,


$$\text{Efficiency }\left(\text{\%}\right)\text{= }\left({\left[\text{FPAb}\right]}_{0}\text{– }\left[\text{FPAb}\right]\right)\text{/ }{\left[\text{FPAb}\right]}_{0}\text{* 100}$$

where [FPAb]_0_ is the initial concentration of FPAb added to the AuNP solution, and [FPAb] is the concentration in the supernatant after centrifugation [36].

The AuNP-FPAbs were also characterised by UV-Vis spectrophotometer, FTIR, Zetasizer, TEM, and AFM [36].

### Detection of snake venom using AuNP-FPAb conjugate

Different concentrations of each snake venom spiked rat plasma were incubated with 10 μL of AuNP-FPAb for 10 min. The colour changes in the solutions were observed in the transparent centrifuge tubes upon adding AuNP-FPAb. The smartphone-based assay was performed by capturing photographs of the tubes using a smartphone and analysing them using ImageJ software. The images were captured with the Motorola Edge 40 Neo, featuring a 50-megapixel camera and a resolution of 1080 x 2400 pixels. The settings for image capture were configured to auto white balance, ISO 400, normal exposure, and the image format was set to JPEG. The average R (red), G (green) and B (blue) colour values from the photographs were measured with the ImageJ image processing toolbox. Photographing of the samples and ImageJ processing of the photographs were carried out in triplicates. Per the Lambert-Beer law equation, the RGB colour values obtained from ImageJ processing were converted to a logarithmic scale to obtain colour intensity in MS Excel 2021.

For actual *in vivo* sample analysis, the plasma collected from the retro-orbital veins of the envenomed rats (s.c.) were treated with 10 μL of AuNP-FPAb for 10 min. The observed colour changes in the solutions were photographed, and the images were analysed as mentioned above.

In order to learn more about the interaction of AuNP-FPAb conjugate and envenomed snake plasma samples, a UV-Vis spectrophotometer (400-700 nm) was used. Additionally, the concentration of venom detected in the envenomed plasma was determined by constructing calibration curves using absorbances recorded from rat plasma samples spiked with snake venom incubated with the AuNP-FPAb conjugates [36].

### Statistical analysis

The results in this study are expressed as mean ± standard deviation (SD) from separate triplicate trials. The differences between control and test values were analysed for significance using the Student’s t-test in Sigma Plot 11.0 for Windows (version 10.0). The significance of differences among several groups was assessed using a one-way analysis of variance (ANOVA), followed by Tukey post hoc analysis in GraphPad Prism software. Statistical significance was determined at p-values ≤ 0.05 and ≤ 0.01.

## Results

### Custom peptides (CPs) designed from major toxins of ‘Big Four’ venomous snakes

For each toxin, two antigenic epitopes were linked via small non-polar glycine (G) to design the CPs with increased antigenic propensity ultimately. The selected toxins, their antigenic epitopes and the custom peptides derived from those epitopes have been summarised in Table 1.

**Table 1:** Antigenic epitopes from major toxins identified in the proteome of ‘Big Four’ venomous snakes of India and their antigenic propensities before and after custom modification.

Name of unique toxin	Antigenic epitopes	Antigenic propensity of epitopes	Epitope-based synthetic peptides. Modified residues are underlined, and the peptide length is shown in parenthesis.	Antigenic propensity of modified peptides	Designation of custom peptide
*Naja naja* (PLA_2_ toxin)	^63^PVDDLDRCCQVHDNC^77^	1.0496	PVDDLDRCCQVHDGGGGNACAASVCDCDRLAAICFAG (37)	1.0819	CP 1
	^110^NACAAAVCDCDRLAAICFAG^130^				
*Daboia russelii* (PLA_2_ toxin)	^40^TDRCCFVHDCCYGNLPDC^57^	1.0453	TDRCCFVHDCCYGNLGGGGENRICECDKAAAICFR (35)	1.0612	CP 2
	^82^ENRICECDKAAAICFR^97^				
*Echis carinatus* (Echicetin toxin)	^51^EEILVDIVVS^60^	1.0285	EEILVDIVVSGGGFRSYEIAIRYSECFVLEKQSVFRTWVATP (42)	1.0636	CP 3
	^91^FFRSYEIAIRYSECFVLEKQSVFRTWVAT^119^				
*Bungarus caeruleus* (Basic phospholipase A_2_ beta- bungarotoxin)	^64^PIDALDRCCYVHDNCYG^80^	1.0536	PIDALDRCCYVHDNCYGGGRRTIICYGAAGTCARIVCDCDRTAALCFGD (49)	1.0736	CP 4
	^101^RRTIICYGAAGTCARIVCDCDRTAALCFGD^130^				
*Bungarus caeruleus* (Basic phospholipase A_2_ KPA2 toxin)	^62^PVDELDRCCYTHD^74^	1.0547	PVDELDRCCYTHDGGGGADTCARFLCDCDRTAAICFASA (39)	1.0591	CP 5
	^108^ADTCARFLCDCDRTAAICFASA^129^				

The KLH-conjugated CPs were used as antigens to raise polyclonal antibodies in rabbits, and then the antibodies were affinity purified to obtain toxin-specific polyclonal antibodies (PAbs). The rabbit antisera showed the presence of a high titre of CP-specific antibodies. The PAbs demonstrated their ability to recognise the CPs with some cross-reactivity among them under identical experimental conditions (S1 Fig). The protein BLAST performed with the CP 1 and CP 4 sequences demonstrated about 90% sequence identity with the PLA_2_ of *N. kaouthia* venom (S1 Data).

### Immunoassays and spectrofluorometric analysis exhibit superior immune recognition of FPAb towards Indian snake venoms as compared to commercial PAV (*in vitro*)

**Fig 1:**
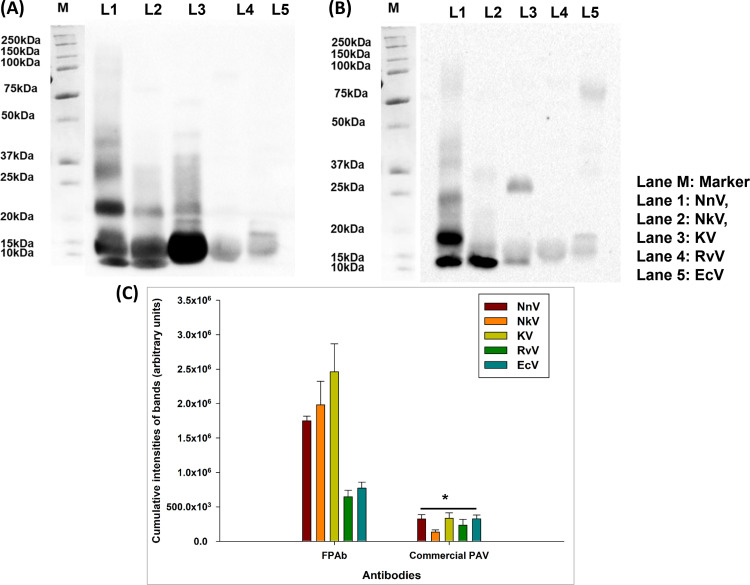
(A) Western blot analysis under reduced conditions to determine the immune-recognition of NnV, NkV, KV, RvV, and EcV by FPAb. Lane 1 represents the immunoblot of NnV, Lane 2 represents the immunoblot of NkV, Lane 3 represents the immunoblot of KV, Lane 4 represents the immunoblot of RvV, Lane 5 represents the immunoblot of EcV, and Lane M denotes the marker. Immunoblot detected by HRP conjugated anti-rabbit IgG. (B) Western blot analysis under reduced conditions to determine the immune-recognition of NnV, NkV, KV, RvV, and EcV by commercial PAV. Lane 1 represents the immunoblot of NnV, Lane 2 represents the immunoblot of NkV, Lane 3 represents the immunoblot of KV, Lane 4 represents the immunoblot of RvV, Lane 5 represents the immunoblot of EcV, and Lane M denotes the marker. Immunoblot detected by HRP conjugated anti-horse IgG. (C) Densitometry analyses of the blot intensities of NnV, NkV, KV, RvV and EcV detected by FPAb and commercial PAV. Significance of difference in immune-recognition of the snake venoms by FPAb with respect to immune-recognition by commercial PAV, *p<0.05. Error bars indicate mean ± SD (n=3).

Western blotting analysis demonstrated the greater immune recognition of the ‘Big Four’ snake venoms and NkV by FPAb, compared to the immune-recognition by commercial PAV (Fig 1A–C and S2 Data). The Ponceau-S red stained PVDF membranes depicted efficient transfer of the venom proteins (S2 Fig).

Dot blot assay conducted to assess the interaction between the secondary antibody and the capture antibody, excluding the antigen, revealed that the secondary antibody (anti-rabbit IgG-HRP/anti-horse IgG-HRP) exhibited diminished immune-recognition of the capture antibody (S3 Fig); these blots will hereafter be referred to as controls without antigen. Moreover, normalizing the dot intensities against the control (excluding antigen intensities), this study revealed the enhanced *in vitro* immune detection of the ‘Big Four’ snake venoms by FPAb in comparison to the identification by individual PAbs, PAbE, and PAbV (S4 Fig).

**Fig 2:**
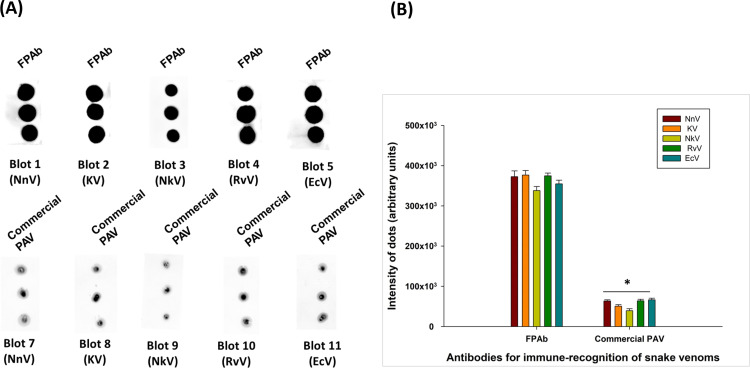
(A) Dot blot assay to determine the immune-recognition of NnV, KV, NkV, RvV, and EcV (1 pg/µL) spiked rat plasma by FPAb and commercial PAV. (B) Image analyses of the dot intensities of immune-recognition by FPAb and commercial PAV were performed using ImageJ software. The dot intensities have been normalized against intensities of control without antigen. Significance of difference immune-recognition of the snake venoms by FPAb with respect to immune-recognition by commercial PAV, *p<0.05. Error bars indicate mean ± SD (n=3).

After normalizing the dot intensities against the control (without antigen intensities), the dot blotting with snake venom spiked rat plasma as antigen exhibited the superior immune cross-reactivity of FPAb towards venom-spiked rat plasma compared to commercial PAV, under identical experimental conditions (Fig 2A and 2B and S3 Data). The FPAb/commercial PAV did not demonstrate any cross-reactivity towards *Mesobuthus tamulus* venom-spiked rat plasma.

**Fig 3:**
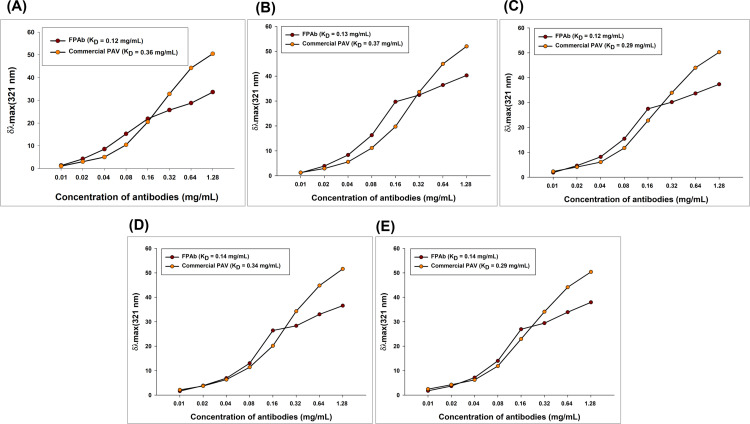
Spectrofluorometric interaction between the snake venoms, FPAb and commercial PAV. The interactions between the fixed concentration of each venom and varying concentrations of FPAb and commercial PAV have been represented in a one-site specific binding curve showing the change in maximum fluorescence intensity (λ_max_) of venom-antibody binding. (A) Interaction of NnV with FPAb and commercial PAV, (B) Interaction of KV with FPAb and commercial PAV, (C) Interaction of NkV with FPAb and commercial PAV, (D) Interaction of RvV with FPAb and commercial PAV, and (E) Interaction of EcV with FPAb and commercial PAV. The graphs were plotted using GraphPad Prism 5.0 software and demonstrate the mean of five scans.

The K_D_ values of the interaction between the snake venoms and FPAb/commercial PAV were determined in the mg protein content of FPAb or commercial PAV/mL [39], and the results elucidated the higher affinity of FPAb towards venom antigens as compared to commercial PAV (less K_D_ value of FPAb compared to commercial PAV against the same venom sample) (Fig 3A–E and S4 Data).

### The FPAb demonstrated better immune-recognition towards the Indian snake venoms as compared to commercial PAV in envenomed rat plasma

**Fig 4:**
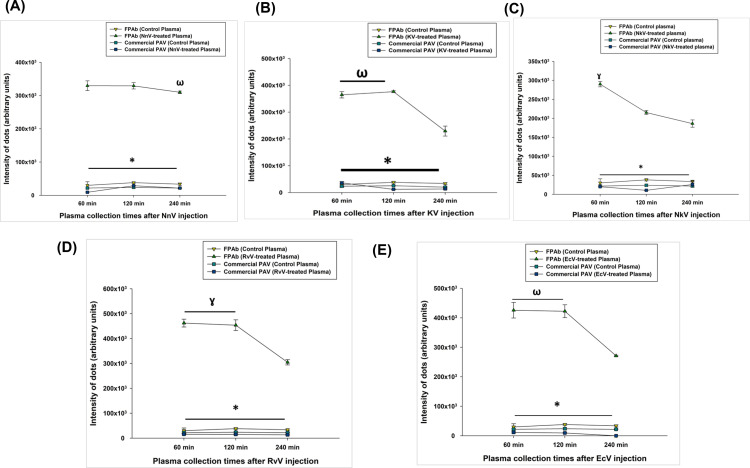
(A) Dot intensities of the immune-recognition of NnV-envenomed rats’ plasma by FPAb and commercial PAV were analysed using ImageJ. Significance of difference in immune-recognition of plasma collected from NnV-envenomed rats (s.c.) at 60 min and 120 min compared to plasma collected from NnV-envenomed rats (s.c.) at 240 min by FPAb, ^ω^p<0.05; immune-recognition of plasma collected from NnV-envenomed rats (s.c.) at 60 min, 120 min and 240 min by FPAb compared to immune-recognition by commercial PAV, *p<0.05; (B) Dot intensities of KV-envenomed rats’ plasma immune-recognised by FPAb and commercial PAV were analysed using ImageJ. Significance of difference in immune-recognition of plasma collected from KV-envenomed rats (s.c.) at 240 min compared to plasma collected from KV-envenomed rats (s.c.) at 60 min and 120 min, ^ω^p<0.05; immune-recognition of plasma collected from KV-envenomed rats (s.c.) at 60 min, 120 min and 240 min by FPAb compared to immune-recognition by commercial PAV, *p<0.05; (C) Dot intensities of NkV-envenomed rats’ plasma immune-recognised by FPAb and commercial PAV were analysed using ImageJ. Significance of difference in immune-recognition of plasma collected from NkV-envenomed rats (s.c.) at 120 min and 240 min compared to plasma collected from NkV-envenomed rats (s.c.) at 60 min, ^ɣ^p<0.05; immune-recognition of plasma collected from NkV-envenomed rats (s.c.) at 60 min, 120 min and 240 min by FPAb compared to immune-recognition by commercial PAV, *p<0.05; (D) Dot intensities of RvV-envenomed rats’ plasma immune-recognised by FPAb and commercial PAV were analysed using ImageJ. Significance of difference in immune-recognition of plasma collected from RvV-envenomed rats (s.c.) at 240 min compared to plasma collected from RvV-envenomed rats (s.c.) at 60 min and 120 min, ^ɣ^p<0.05; immune-recognition of plasma collected from RvV-envenomed rats (s.c.) at 60 min, 120 min and 240 min by FPAb compared to immune-recognition by commercial PAV, *p<0.05; (E) Dot intensities of EcV-envenomed rats’ plasma immune-recognised by FPAb and commercial PAV were analysed using ImageJ. Significance of difference in immune-recognition of plasma collected from EcV-envenomed rats (s.c.) at 240 min compared to plasma collected from EcV-envenomed rats (s.c.) at 240 min at 60 min and 120 min, ^ω^p<0.05; immune-recognition of plasma collected at 60 min, 120 min and 240 min by FPAb compared to recognition by commercial PAV, *p<0.05. Error bars indicate mean ± SD (n=3).

Dot blot analysis depicted the time-dependent immune-recognition of the snake venoms by FPAb in the plasma of subcutaneously envenomed Wistar rats. In NnV envenomed rats (group 2), no significant difference was found in the venom recognition by FPAb between 60- and 120-min post-injection; however, a slight decline in immune recognition was observed at 240 min (Figs 4A and S5A and S5 Data). Maximum venom recognition of KV in group 3 rats’ plasma by FPAb was observed at 60- and 120-min post-injection, with a decline of immune recognition at 240 min (Figs 4B and S5B and S5 Data). The FPAb showed the highest detection of NkV in plasma (group 6) at 60 min post-injection; however, the immune recognition of FPAb demonstrated a gradual decline at 120- and 240-min post-injection NkV plasma (Figs 4C and S5C and S5 Data).

For the rats injected with RvV subcutaneously (group 4), the maximum RvV recognition was observed at 60- and 120-min post-injection, and the recognition declined significantly at 240 min (Figs 4D and S5D and S5 Data). The highest EcV recognition (group 5) was observed between 60- and 120-min post-injection, and after that, a sharp decline in the intensity of recognition was observed (Figs 4E and S5E and S5 Data). However, the intensity of recognition of venom from the Viperidae family of snakes was higher (p<0.01) than the Elapidae family of snakes. Notably, since the antibodies constituting the FPAb were raised against antigenic peptides synthesised using the toxins of the ‘Big Four’ venomous snakes of India, it may have a lower affinity towards NkV; therefore, their detection in plasma may be lesser than the ‘Big Four’ snake venoms.

In all the cases, dot blot analysis demonstrated the superior immune recognition of tested snake venoms by FPAb compared to commercial PAV raised against native toxins, under identical experimental conditions (Fig 4A–E and S5 Data).

### Characterisation of FPAb conjugated AuNPs

**Fig 5:**
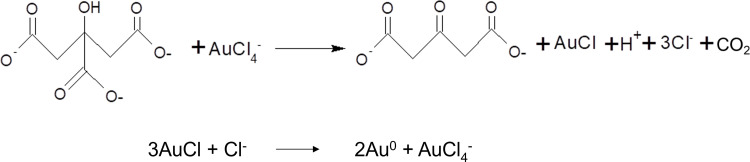
Chemical reaction sequence for citrate reduction-based AuNP synthesis.

The production of gold nanoparticles (AuNPs) using citrate reduction involves the utilisation of citrate to aid the reduction of gold (III) to gold (0), hence imposing growth limitations on the AuNPs [44–46]. The following scheme depicts the reaction during citrate reduction-based AuNP synthesis (Fig 5).

The AuNPs synthesised by this method exhibited a wine-red colour, and their UV-Vis spectra (400-700 nm) depicted a plasmonic peak at around 527 nm. After the conjugation of FPAb to the citrate-capped AuNPs, the plasmonic peak showed a bathochromic shift to 537 nm (S6A Fig).

FTIR presented an infrared spectrum of AuNP with peaks at 3368 cm^−1^, 1567 cm^−1^, 1475 cm^−1,^ and 1399 cm^−1,^ characteristic of –O–H stretching, –C=O stretching, and –C–H bending, respectively, and indicate the formation of citrate-stabilised AuNPs [47]. After conjugation of FPAb to AuNP, the peaks associated with –O–H stretching and –C=O stretching disappeared, and new peaks appeared at 1622 cm^−1^ and 1532 cm^−1^ which correspond to primary amide NH_2_ bending and secondary amide N–H bending under the amide II band, respectively [47] (S6B Fig).

The citrate-capped AuNPs and AuNP-FPAb conjugate recorded zeta potential (mV) of −34.5 ± 0.173 mV and −26.8 ± 0.02 mV, respectively (S6C Fig).

The TEM analysis performed in this study showed the synthesis of monodisperse citrate-capped AuNPs, which were aggregated after conjugation to FPAb (S7A and S7B Fig). From the images obtained, the average diameter of the AuNP and AuNP-FPAb conjugate particles was determined at 18.99 ± 0.41 nm and 37.52 ± 0.99 nm, respectively (S7C and S7D Fig). The AFM analysis further demonstrated the synthesis and conjugation of AuNPs performed (S8A and S8B Fig), which depicted that the citrate-capped AuNPs and AuNP-FPAb conjugate particles measured their heights at 18.53 ± 0.26 nm and 36.21 ± 0.45 nm, respectively (S8C and S8D Fig). The adsorption efficiency of FPAb to AuNP was determined at 59.3% (S9 Fig).

### Detection of Indian snake venoms in envenomed Wistar strain rats (*in vivo*) by AuNP-FPAb conjugate using digital image colourimetry

Snake venoms spiked in rat plasma were detected using smartphone images. From the images recorded, the average R (red), G (green), and B (blue) values were analysed using ImageJ. The RGB colour values obtained were converted to a logarithmic scale to get the colour intensity according to the Lambert-Beer law equation [33,48]:


$$\text{Colour Intensity of Red }\left({\text{I}}_{\text{R}}\right)\text{= log }\left({\text{R}}_{\text{0}}{\text{/R}}_{\text{S}}\right)\text{,}$$ (1)

$$\text{Colour Intensity of Green }\left({\text{I}}_{\text{G}}\right)\text{= log }\left({\text{G}}_{\text{0}}{\text{/G}}_{\text{S}}\right)\text{,}$$ (2)

$$\text{Colour Intensity of Blue }\left({\text{I}}_{\text{B}}\right)\text{= log }\left({\text{B}}_{\text{0}}{\text{/B}}_{\text{S}}\right)\text{,}$$ (3)

The average blank and sample colour values are R_0_, G_0_, B_0_, R_S_, G_S_, and B_S_, respectively.

**Fig 6:**
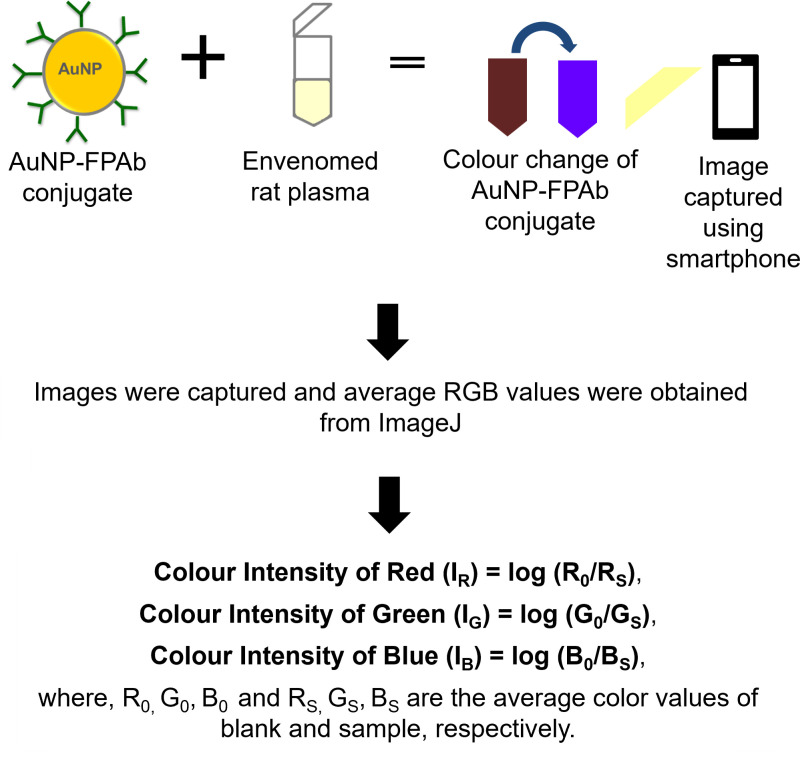
Schematic illustrating the colorimetric assay by determining colour intensities using RGB values from the smartphone images of interaction between AuNP-FPAb conjugate and venom-treated plasma. Prepared using MS PowerPoint 2021.

**Fig 7:**
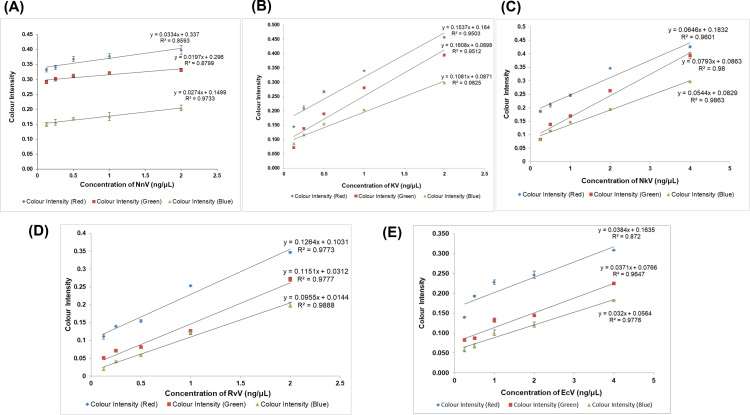
Linear fitted plot based on the relationship between colour intensities obtained from RGB of smartphone images and different concentrations of snake venom spiked rat plasma. (A) NnV concentrations of 0.125-2 ng/μL, (B) KV concentrations of 0.125-2 ng/μL, (C) NkV concentrations of 0.25-4 ng/μL, (D) RvV concentrations of 0.125-2 ng/μL, (E) EcV concentrations of 0.25-4 ng/μL. Error bars indicate mean ± SD (n=3).

Fig 6 illustrates the schematic for a colourimetric assay that determines colour intensities by utilizing the RGB values obtained from smartphone images of the interaction between AuNP-FPAb conjugate and venom-treated plasma. Linear regression standard curves were prepared from the colour intensities obtained from each venom-spiked plasma (Fig 7A–E and S6 Data). From the equations and R^2^ values depicted in Fig 7A–E, the blue (B) colour showed the highest linearity as compared to the red (R) and green (G) colours for all the snake venoms studied. This observation may be due to the change in colour of AuNP-FPAb conjugates from burgundy to blue upon binding with venoms.

From the equations obtained from the linear regression curves (Fig 7A–E), the limit of detection (LOD) for blue colour was determined to be 330 pg/μL (NnV), 240 pg/μL (KV), 210 pg/μL (RvV), 620 pg/μL (EcV), and 430 pg/μL (NkV). The LOD was determined using the formula 3.3 σ/S, where σ is the standard deviation of the response, and S is the slope of the calibration curve.

**Fig 8:**
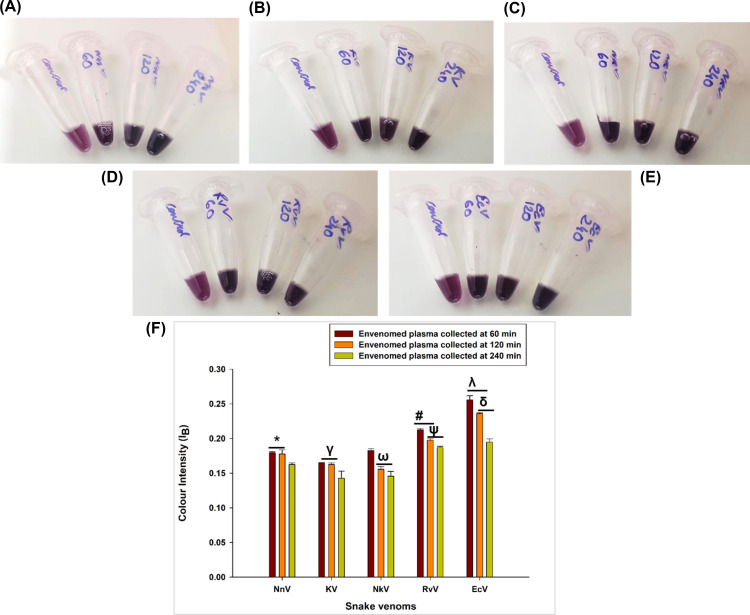
Colour changes of AuNP-FPAbs in the presence of control and venom-treated rat plasma. In case of control plasma, the AuNP-FPAb conjugate’s colour is violet-pink, while in case of the venom-treated plasmas, the colour of the AuNP-FPAb conjugate’s changes to blue-grey due to aggregation of the AuNP-FPAb conjugates. Photographs taken by Upasana Puzari (First author). (A) NnV-envenomed rat plasma collected at 60 min, 120 min and 240 min, (B) KV-envenomed rat plasma collected at 60 min, 120 min and 240 min, (C) NkV-envenomed rat plasma collected at 60 min, 120 min and 240 min, (D) RvV-envenomed rat plasma collected at 60 min, 120 min and 240 min, (E) EcV-envenomed rat plasma collected at 60 min, 120 min and 240 min, (F) Blue colour intensities (I_B_) of all the snake venom-treated rat plasmas compared to the control plasma. Significance of difference for I_B_ of NnV-treated plasma collected at 60 and 120 min compared to I_B_ of NnV-treated plasma collected at 240 min, *p<0.05; I_B_ of KV-treated plasma collected at 60 and 120 min compared to I_B_ of KV-treated plasma collected at 240 min, ^γ^p<0.05; I_B_ of NkV-treated plasma collected at 120 and 240 min compared to I_B_ of NkV-treated plasma collected at 60 min, ^ω^p<0.05; I_B_ of RvV-treated plasma collected at 60 and 120 min compared to I_B_ of RvV-treated plasma collected at 240 min, ^#^p<0.05; I_B_ of RvV-treated plasma collected at 120 and 240 min compared to I_B_ of RvV-treated plasma collected at 60 min, ^ψ^p<0.05; I_B_ of EcV-treated plasma collected at 60 and 120 min compared to I_B_ of EcV-treated plasma collected at 240 min, ^λ^p<0.05; I_B_ of EcV-treated plasma collected at 120 and 240 min compared to I_B_ of EcV-treated plasma collected at 60 min, ^δ^p<0.05. Error bars indicate mean ± SD (n=3).

The plasma samples obtained from the retro-orbital blood collected from the subcutaneously envenomed rat plasma samples were detected with AuNP-FPAbs, and the images recorded were analysed by ImageJ software (Fig 8A–F and S7 Data). The I_B_ was calculated from the images of time-dependent plasma collected for all the snake venoms studied, the quantity of venom in plasma was calculated using equation 4 as shown below and the results have been summarized in Table 2. The alterations in colour resulting from the interaction between the envenomed plasmas and AuNP-FPAb conjugates were measured as absorbance with a UV–Vis spectrophotometer (S10 Fig). Calibration curves were systematically prepared for each of the snake venom spiked plasmas (S11 Fig), and the resulting equations were employed to quantify the amount of snake venom detected in the envenomed plasmas (Table 2).

**Table 2:** Determination of blue colour intensity and quantification of venom in envenomed rat plasma using Digital Image Colourimetry and UV-Vis spectrophotometer.

Plasma from rats envenomed with Indian snake venoms collection	Time of blood collection (min)	Average I_B_	Quantity of venom in plasma calculated using I_B_ (ng/µL)	Quantity of venom in plasma calculated using I_B_ (in percent of venom found in plasma)	Quantity of venom in plasma calculated using absorbance (ng/µL)	Quantity of venom in plasma calculated using absorbance (in percent of venom found in plasma)
**Indian cobra venom (NnV)**	60	0.18	1.09	26.5	0.99	24.0
	120	0.17	1.01	24.6	0.68	16.5
	240	0.16	0.47	11.4	0.38	9.3
**Indian krait venom (KV)**	60	0.16	0.75	31.9	0.81	34.3
	120	0.16	0.65	27.7	0.70	29.9
	240	0.13	0.44	18.7	0.42	18.0
**Indian monocled cobra venom (NkV)**	60	0.19	1.85	22.5	2.46	29.9
	120	0.15	1.30	15.8	1.59	19.4
	240	0.14	1.11	13.5	1.48	18.0
**Indian russell’s viper venom (RvV)**	60	0.21	2.07	50.4	2.35	57.4
	120	0.20	1.91	46.5	2.33	56.8
	240	0.19	1.81	44.0	2.24	54.2
**Indian saw-scaled viper venom (EcV)**	60	0.26	6.36	77.5	1.57	66.7
	120	0.24	5.74	70.0	1.47	62.6
	240	0.20	4.33	52.7	1.43	60.6


$$\text{Quantity of venom in plasma calculated using IB/absorbance }\left(\text{in percent of venom found in plasma}\right)\text{= Quantity of venom in plasma calculated using linear regression curve of IB/absorbance }\left(\text{ng/µL}\right)\text{/ Amount of venom injected in 220 g rat }\left(\text{total blood volume in 220 g rat assumed to be 15 mL}\right)\left(\text{ng/µL}\right)\text{*100 }$$ (4)

From Table 2, quantity of venom in envenomed plasma obtained from both Digital Image Colourimetry and UV-Vis spectrophotometry was found to be comparable.

## Discussion

This study describes the potential of a toxin-specific polyclonal antibody formulation to differentiate between bite cases with and without snake envenomation, commonly known as wet and dry snake bite, respectively, among India’s medically critical venomous snakes. The produced polyclonal antibodies were combined in a specific ratio to create the antibody formulation, FPAb, which exhibited synergistic immune recognition of the venoms under both *in vitro* and *in vivo* conditions. Detection of Indian snake venoms in envenomed rat plasma using the antibody formulation FPAb has been carried out using digital image colourimetry method using AuNPs. Thus, this method may have potential to confirm venomous snakebites in samples for accurate diagnosis of envenomation.

Numerous researchers have employed various assays for developing tests or procedures for detecting snake envenomation from venomous snakes. In 2017, Shaikh et al. introduced the Venom Detection ELISA Test (VDET), a device utilizing dot-blot ELISA with plasma obtained from mice envenomed 60 minutes after venom injection. This test provides a binary result regarding envenomation by the Indian ‘Big Four’ venomous snakes and can be completed in 20-25 min, with a limit of detection of 1 ng/mL [14]. A recent study investigated the use of infrared thermal imaging to distinguish between venomous snakebites and non-venomous or dry bites in patients [49]. Most patients exhibiting signs of local envenomation, whether accompanied by systemic envenomation or not, displayed temperature alterations. They obtained infrared photos in still image mode via a thermal imaging camera connected to an Android smartphone. The photos were subsequently analysed to ascertain the condition of envenomation [49]. Our work suggests a quick detection approach that may identify the presence of venom in plasma within 10 min, allowing one to distinguish between wet and dry snakebite cases. Furthermore, the antibody formulation described in our work demonstrated the detection of snake venoms in plasma up to 240 min after venom injection, which was not proven in the VDET trial. Though the LOD described in the current work is lower than the stated LOD of the VDET study, future investigations using monoclonal antibodies may boost the sensitivity of the current approach even further.

Additionally, two Indian patents for developing snake venom detection kits for Indian’ Big Four’ snake venoms have been granted [50, 51]. The inventors of a specific patent have developed a method for producing monospecific antibodies, which exhibit no cross-reactivity with any of the other three species, and bispecific antibodies, which show no cross-reactivity with any of the other two species. This is an *in vitro* method that targets the Indian ‘Big Four’ snake venoms and includes the creation of lateral flow immunoassay devices that utilize the generated antibodies [51]. A separate patent introduced a two-site ELISA-based kit and a lateral flow immunoassay for detecting the venoms of the ‘Big Four’ Indian snakes in clinical samples from snakebite victims [50].

Toxins from families of PLA_2_, snake venom metalloprotease, snake venom serine protease and three-finger toxins are generally dominant in the venom composition of all snake species [52]. Since developing a species-specific snake venom detection method necessitates targeting toxins from these families, there is a concern regarding a high degree of cross-reactivity between snake venom toxins of the same or heterologous family [6,39,53–55]. This reason has proven to be a hurdle in developing detection devices that may differentiate between genera/species of venomous snakes. Although numerous attempts have been put forward for detecting Indian snake venoms using various analytical methods like antigen-antibody interaction, aptamers, etc., none of them has been translated and commercialised for use in a clinical setting [12,14,17,50,51,56].

In recent years, the design and usage of synthetic peptides that mimic selected protein regions have seen a potential increase [36,57–60]. In this study, to circumvent the problem of using whole venom or purified venom-toxins as immunogens for the production of polyclonal antibodies, we have synthesised custom peptide immunogens designed using antigenic sites with highest antigenic propensity from the major toxins like PLA_2_ and snaclec of Indian ‘Big Four’ venomous snakes. These antigenic sites were further modified to enhance their antigenic propensity, producing high antibody titre against these peptides. Generally, to raise high-titre polyclonal antibodies, it is necessary to conjugate the peptides to a carrier protein such as KLH, bovine serum albumin (BSA), or ovalbumin [57,61,62]. Therefore, we conjugated the custom peptides with KLH in our study, ultimately producing high-titre polyclonal antibodies in rabbit serum.

The current study has used immunoblotting techniques to demonstrate the ability of toxin-specific antibody formulation, FPAb, to detect the presence of a picogram quantity of venom from Indian snakes in the body fluids of envenomed animals. Our study elucidates better immune-recognition by FPAb than commercial PAV for Indian snake venoms at a low 1 pg/μL concentration, under *in vitro* conditions. The observed effect may happen because FPAb comprises antibodies against the antigenic custom peptides designed explicitly from ‘Big Four’ snakes’ low-molecular-mass toxins (PLA_2_ and snaclec). On the contrary, inferior immune-recognition for the venoms by commercial PAV may be because they are raised against whole venom of the ‘Big Four’ snakes and do not contain sufficient antibodies against the pharmacologically active, weakly immunogenic proteins in these venoms [21,23–25,63,64]. Given the ability of FPAb to detect the venom of ‘Big Four’ venomous snakes and *N. kaouthia,* under *in vitro* conditions, we have explored the potential of FPAb for detecting these venoms in animal plasma.

Animal models are crucial in experimental research (pre-clinical study) for refining diagnostic techniques and novel drug therapeutics before clinical trials [65]. In pharmacology and toxicology studies, experimental rats are generally preferred over mice as they share a similar toxin eradication pathway as humans [66]. Additionally, the larger body size of rats allows serial blood draws and sampling over time [67]. Therefore, our study has experimentally envenomed Wistar strain albino rats with the venoms of India’s ‘Big Four’ venomous snakes and *N. kaouthia.* Compared to commercial PAV, FPAb better recognised the venom in plasma collected from the envenomed rats at regular intervals.

Generally, snakes envenom their victims by injecting their venoms mostly subcutaneously or occasionally intramuscularly, pending the release of the toxins into the interstitial space [68–70]. Studies have revealed that snake venom injected into patients gets absorbed quickly from the bloodstream, binding very rapidly with their target organs or specific receptors, thereby lessening the amount of venom left in systemic circulation for being detected by the diagnostic kit [6,71]. Snake venom in circulation may exhibit two phases: a quick distribution phase (within 60 min) and a prolonged elimination phase (12-24 h) [29,72–74]. Our study corroborates this observation, and we have demonstrated that immune recognition of the snake venoms in the envenomed plasma by FPAb decreased as time elapsed post-injection, i.e., venom detected in plasma at 240 min post-injection is lesser than at 60- and 120-min post-venom injections.

It is known that agglomeration of AuNP-FPAb conjugates upon interaction of venoms could result in a colour change. This property of AuNP was used in this study to demonstrate a colourimetric detection and quantification of the Indian Big Four venomous snakes and *N. kaouthia* in envenomed rat plasma*.*

The formation of AuNPs and AuNP-FPAb bioconjugates was first confirmed using a few biophysical techniques. The size of the AuNP and AuNP-FPAb particles were determined to be about 18 nm and 37 nm, respectively, indicating the binding of antibodies to AuNP. It was observed that when envenomed plasmas from the rats were incubated with the AuNP-FPAb conjugates, the colour of the solution changed from violet-pink to blue-grey colour within 5-10 min due to agglomeration [75, 76]. Recent breakthroughs in the smartphone technology field have improved their usage to satisfy the needs of many scientific fields [32,77,78]. Thus, digital image colourimetry based on smartphone images could differentiate between control (non-envenomed) plasma and envenomed plasma and quantify the venom present in envenomed plasma during the studied post-injection time duration. This method of detection will help detect envenomation in the health centres of remote villages where sophisticated instrumental facilities are not available. The venom quantity detected by FPAb in the plasma decreased as the time post-injection increased. Notably, since the antibodies constituting the FPAb were raised against antigenic peptides synthesised using the toxins of the ‘Big Four’ venomous snakes of India, it may have a lower affinity towards NkV and therefore, their detection in plasma may be lesser than the ‘Big Four’ snake venoms; however, the sequence similarity observed between the CP 1, CP 4 and *N. kaouthia* PLA_2_ justify its venom detection by FPAb. An additional noteworthy finding was that the intensity of blue colour in the plasma treated with Viperidae venoms (RvV and EcV) was greater than that in the plasma treated with Elapidae venoms (NnV, KV, and NkV). Thus, more venom could be identified in the plasma of rats treated with Viperidae venom at a particular moment compared to those treated with Elapidae venom. The observed phenomenon can be attributed to the fast attachment of the Elapid venoms, abundant in low molecular weight toxins, to their specific tissue targets. As a result, these venoms are promptly eliminated from the bloodstream. In contrast, Viperid venoms containing high-molecular-weight toxins are absorbed gradually from the injection site via the lymphatic system, resulting in prolonged and heightened presence in the bloodstream [70,79,80].

Snakebite cases are mostly occupational health hazard and affect rural population of the underdeveloped and developing countries [81]. Reports from India have suggested that majority of the snakebite deaths have been recorded in rural areas (97%) during monsoon months [81]. However, the unofficial count of morbidity and mortality due to snake envenomation may be far higher due to poor documentation [6]. In clinical settings, it is crucial to determine whether a snakebite is wet or dry since this knowledge informs the right medical actions, such as deciding whether antivenom is required. [82]. The World Health Organization (WHO) has stated that snakebite management mainly relies on identifying snake species, clinical diagnosis, and the appropriate administration of antivenoms. The WHO recommendations emphasize the necessity for commercial tests that confirm snake envenomation, enabling doctors to select and deliver the optimum amount of antivenoms for patient treatment [83]. This study proposes a portable smartphone-based colourimetric method for detecting and quantifying venom from the Indian ‘Big Four’ snakes, which is essential for antivenom therapy, potentially leading to developing a device for on-site assessment of envenomation in patients.

The snake envenomation detection approach presented in this paper may enable medical professionals to ascertain if a snakebite is venomous or non-venomous, enhancing hospital care of snake envenomation cases. Nevertheless, other constraints must be resolved before using this technology in a clinical context. The current detection approach necessitates clinical testing using bodily fluid samples from victims of snake envenomation. The clinical study must incorporate an adequate sample size of actual victim samples to validate the applicability of FPAb for detection purposes in clinical settings. Since the proposed method is a proof-of-concept, future investigations may be designed to refine antibody raising, and monoclonal antibodies raised against the snake venom toxins may be considered to develop a particular and sensitive detection method. The smartphone-based detection system presented in this work requires improvements in real-time result processing, interpretation, and communication to necessary staff before it can be used in rural clinics.

This study presents a straightforward and cost-effective method that could aid in developing kits for treating venomous snakebites in patients. The research indicates the generation of polyclonal antibodies targeting toxins from India’s four most venomous snakes. The antibodies generated were combined in a specific ratio to illustrate synergistic immune-recognition of India’s ‘Big Four’ venomous snakes and *N. kaouthia* venoms, both *in vitro* and *in vivo*. Digital image colourimetry enhanced the detection by the antibody formulation. Further research is necessary to validate the suitability of this detection method using envenomed patient plasma and other body fluids in both field and clinical settings.

## Supporting information

S1 Text Methodologies for supporting information.
Methodologies for: KLH conjugation of the CPs, Raising and purifying custom peptide-specific antibodies by immunising rabbits with KLH-conjugated CPs and Synthesis of AuNP-FPAb conjugates.
(DOCX)

S1 Fig **(A)** Dot blot assay to determine the immune-recognition of individual PAbs towards the CPs. Dot blot intensities of immune recognition demonstrated by the individual PAbs towards the CPs. **(B)** PAb 1 demonstrated cross-reactivity towards CP2-5. Significance of difference in recognition of PAb 1 towards CP2, CP3, CP4 and CP5 compared to CP1, *p<0.05. **(C)** PAb 2 demonstrated some cross-reactivity towards CP3 and CP4. Significance of difference in recognition of PAb 2 towards CP3 and CP4 compared to CP2, ^ɣ^p<0.05. **(D)** PAb 3 demonstrated cross-reactivity towards CP2, CP4 and CP5. Significance of difference in recognition of PAb 3 towards CP2, CP4 and CP5 compared to CP3, ^ω^p<0.05. **(E)** PAb 4 demonstrated specificity towards CP4. **(F)** PAb 5 demonstrated cross-reactivity towards CP4. Significance of difference in recognition of PAb 5 towards CP4 compared to CP5, ^ψ^p<0.05. Error bars indicate mean ± SD (n=3).
(TIFF)

S2 Fig **(A, B)** Ponceau-S red stained blots indicating snake venom protein transfer.
(TIF)

S3 Fig **(A)** Dot blot assay to determine immune-recognition of PAbs (individual PAb 1, 2, 3, 4, 5, PAbE, PAbV and FPAb) using anti-rabbit IgG-HRP and commercial PAV using anti-horse IgG-HRP; **(B)** Dot intensities of the immune-recognition demonstrated by the secondary antibodies as stated in (A). Significance of difference immune-recognition of FPAb by anti-rabbit IgG-HRP with respect to immune recognition of individual PAbs, PAbE, PAbV by anti-rabbit IgG-HRP and commercial PAV by anti-horse IgG-HRP, *p<0.05. Error bars indicate mean ± SD (n=3).
(TIF)

S4 Fig **(A)** Dot blot assay to determine immune-recognition of PAbs (individual PAb 1, 2, 3, 4, 5, PAbE, PAbV and FPAb) towards NnV, KV, NkV, RvV and EcV (1 pg/μL)**, (B)** Dot intensities of the immune-recognition of NnV, KV, NkV, RvV and EcV by individual PAbs, PAbE, PAbV and FPAb. Significance of difference in immune-recognition of NnV, KV, NkV, RvV and EcV by PAb 1,2,3,4,5, PAbE and PAbV compared to immune-recognition by FPAb *p<0.05. Error bars indicate mean ± SD (n=3).
(TIFF)

S5 Fig **(A)** Dot blot assay to determine immune-recognition of NnV in the plasma of the group 1 and 2 rats by FPAb and commercial PAV when the blood was collected at 60 min, 120 min, and 240 min post-injection (s.c.). Blots 1-3 incubated with control plasma (s.c.) collected after 60 min, 120 min, and 240 min recognised by FPAb; Blots 4-6 incubated with NnV -treated plasma (s.c.) collected after 60 min, 120 min, and 240 min recognised by FPAb; Blots 7-9 incubated with control plasma (s.c.) collected after 60 min, 120 min, and 240 min recognised by commercial PAV; Blots 10-12 incubated with NnV -treated plasma (s.c.) collected after 60 min, 120 min, and 240 min recognised by commercial PAV. **(B)** Dot blot assay to determine immune-recognition of KV in the plasma of the group 1 and 3 rats by FPAb and commercial PAV when the blood was collected at 60 min, 120 min, and 240 min post-injection (s.c.). Blots 1-3 incubated with control plasma (s.c.) collected after 60 min, 120 min, and 240 min recognised by FPAb; Blots 4-6 incubated with KV-treated plasma (s.c.) collected after 60 min, 120 min, and 240 min recognised by FPAb; Blots 7-9 incubated with control plasma (s.c.) collected after 60 min, 120 min, and 240 min recognised by commercial PAV; Blots 10-12 incubated with KV-treated plasma (s.c.) collected after 60 min, 120 min, and 240 min recognised by commercial PAV. **(C)** Dot blot assay to determine immune-recognition of NkV in the plasma of the group 1 and 6 rats by FPAb and commercial PAV when the blood was collected at 60 min, 120 min, and 240 min post-injection (s.c.). Blots 1-3 incubated with control plasma (s.c.) collected after 60 min, 120 min, and 240 min recognised by FPAb; Blots 4-6 incubated with NkV-treated plasma (s.c.) collected after 60 min, 120 min, and 240 min recognised by FPAb; Blots 7-9 incubated with control plasma (s.c.) collected after 60 min, 120 min, and 240 min recognised by commercial PAV; Blots 10-12 incubated with NkV-treated plasma (s.c.) collected after 60 min, 120 min, and 240 min recognised by commercial PAV. **(D)** Dot blot assay to determine immune-recognition of RvV in the plasma of the group 1 and 4 rats by FPAb and commercial PAV when the blood was collected at 60 min, 120 min, and 240 min post-injection (s.c.). Blots 1-3 incubated with control plasma (s.c.) collected after 60 min, 120 min, and 240 min recognised by FPAb; Blots 4-6 incubated with RvV-treated plasma (s.c.) collected after 60 min, 120 min, and 240 min recognised by FPAb; Blots 7-9 incubated with control plasma (s.c.) collected after 60 min, 120 min, and 240 min recognised by commercial PAV; Blots 10-12 incubated with RvV-treated plasma (s.c.) collected after 60 min, 120 min, and 240 min recognised by commercial PAV. **(E)** Dot blot assay to determine immune-recognition of EcV in the plasma of the group 1 and 5 rats by FPAb and commercial PAV when the blood was collected at 60 min, 120 min, and 240 min post-injection (s.c.). Blots 1-3 incubated with control plasma (s.c.) collected after 60 min, 120 min, and 240 min recognised by FPAb; Blots 4-6 incubated with EcV-treated plasma (s.c.) collected after 60 min, 120 min, and 240 min recognised by FPAb; Blots 7-9 incubated with control plasma (s.c.) collected after 60 min, 120 min, and 240 min recognised by commercial PAV; Blots 10-12 incubated with EcV-treated plasma (s.c.) collected after 60 min, 120 min, and 240 min recognised by commercial PAV.
(TIFF)

S6 Fig **(A)** UV-Vis spectra depicting AuNP and AuNP-FPAb conjugate. The absorbance is the mean of values obtained in triplicates; (B) FTIR spectra of AuNP and AuNP-FPAb conjugate; (C) Zeta potential of AuNP and AuNP-FPAb conjugate.
(TIF)

S7 Fig TEM images of **(A)** AuNP and **(B)** AuNP-FPAb conjugate particle at 20 nm magnification; Histogram depicting Particle size distribution of **(C)** AuNP and **(D)** AuNP- FPAb conjugate particle in TEM images, with Gaussian function, fit using Originpro 8.5.
(TIF)

S8 Fig Topographic 2D AFM images with scanned area 1000 x 1000 nm of **(A)** AuNP, **(B)** AuNP-FPAb conjugate; Histogram of height distribution of **(C)** AuNP, **(D)** AuNP-FPAb conjugate, from the topographic 2D AFM images with scanned area 1000 x 1000 nm.
(TIF)

S9 Fig Calibration curve for estimating FPAb left in the supernatant after AuNP-conjugation. Error bars indicate mean ± SD (n=3).
(TIF)

S10 Fig **(A)** Absorbance spectra of the AuNP-FPAb conjugate in the presence of control (untreated, group 1 rats) and NnV-treated plasma collected at 60 min, 120 min and 240 min post-injection (group 2 rats). The absorption maximum (λ_max_) for Control plasma was at 537 nm. On interacting with the envenomed plasma, the *λ*_max_ shifted to 630 nm, 602 nm and 580 nm for NnV-treated plasma collected at 60 min, 120 min and 240 min, respectively.; **(B)** Absorbance spectra of the AuNP-FPAb conjugate in the presence of control (untreated, group 1 rats) and KV-treated plasma collected at 60 min, 120 min and 240 min post-injection (group 3 rats). The absorption maximum (*λ*_max_) for Control plasma was at 537 nm. On interacting with the envenomed plasma, the *λ*_max_ shifted to 610 nm, 589 nm and 555 nm for KV-treated plasma collected at 60 min, 120 min and 240 min, respectively.; **(C)** Absorbance spectra of the AuNP-FPAb conjugate in the presence of control (untreated, group 1 rats) and NkV-treated plasma collected at 60 min, 120 min and 240 min post-injection (group 6 rats). The absorption maximum (*λ*_max_) for Control plasma was at 537 nm. On interacting with the envenomed plasma, the *λ*_max_ shifted to 604 nm, 548 nm and 546 nm for NkV-treated plasma collected at 60 min, 120 min and 240 min, respectively.; **(D)** Absorbance spectra of the AuNP-FPAb conjugate in the presence of control (untreated, group 1 rats) and RvV-treated plasma collected at 60 min, 120 min and 240 min post-injection (group 4 rats). The absorption maximum (*λ*_max_) for Control plasma was at 537 nm. On interacting with the envenomed plasma, the *λ*_max_ shifted to 548 nm, 547 nm and 543 nm for RvV-treated plasma collected at 60 min, 120 min and 240 min, respectively.; **(E)** Absorbance spectra of the AuNP-FPAb conjugate in the presence of control (untreated, group 1 rats) and EcV-treated plasma collected at 60 min, 120 min and 240 min post-injection (group 5 rats). The absorption maximum (*λ*_max_) for Control plasma was at 537 nm. On interacting with the envenomed plasma, the *λ*_max_ shifted to 550 nm, 548 nm and 547 nm for EcV-treated plasma collected at 60 min, 120 min and 240 min, respectively.
(TIF)

S11 Fig **(A)** Absorbance spectrum for NnV spiked rat plasma detection by AuNP-FPAb conjugate. Absorbance curves correspond to plasma samples containing 0.125-2 ng/μL NnV; **(B)** Calibration curve for NnV spiked rat plasma detection at concentrations 0.125-2 ng/μL; **(C)** Absorbance spectrum for KV spiked rat plasma detection by AuNP-FPAb conjugate. Absorbance curves correspond to plasma samples containing 0.125-2 ng/μL KV; **(D)** Calibration curve for KV spiked rat plasma detection at concentrations 0.125-2 ng/μL; **(E)** Absorbance spectrum for NkV spiked rat plasma detection by AuNP-FPAb conjugate. Absorbance curves correspond to plasma samples containing 0.25-4 ng/μL NkV; **(F)** Calibration curve for NkV spiked rat plasma detection at concentrations 0.25-4 ng/μL; **(G)** Absorbance spectrum for RvV spiked rat plasma detection by AuNP-FPAb conjugate. Absorbance curves correspond to plasma samples containing 0.125-2 ng/μL RvV; **(H)** Calibration curve for RvV spiked rat plasma detection at concentrations 0.125-2 ng/μL; **(I)** Absorbance spectrum for EcV spiked rat plasma detection by AuNP-FPAb conjugate. Absorbance curves correspond to plasma samples containing 0.25-4 ng/μL EcV; **(J)** Calibration curve for EcV spiked rat plasma detection at concentrations 0.25-4 ng/μL; Error bars indicate mean ± S.D. (n = 3).
(TIFF)

S1 Data Multiple sequence alignment of CPs and *Naja kaouthia* PLA_2_. (TXT)

S2 Data Spreadsheet containing data for Fig 1. (XLSX)

S3 Data Spreadsheet containing data for Fig 2. (XLSX)

S4 Data Spreadsheet containing data for Fig 3. (XLSX)

S5 Data Spreadsheet containing data for Fig 4. (XLSX)

S6 Data Spreadsheet containing data for Fig 7. (XLSX)

S7 Data Spreadsheet containing data for Fig 8F. (XLSX)

## References

[1] WHO. WHO Expert Committee on Biological Standardization: Seventy fifth report. Geneva; 2022.

[2] Menon JC, Bharti OK, Dhaliwal RS, John D, Menon GR, Grover A, et al. ICMR task force project- survey of the incidence, mortality, morbidity and socio-economic burden of snakebite in India: A study protocol. PLoS One. 2022;17(8):e0270735. doi: 10.1371/journal.pone.0270735 35994445 PMC9394808

[3] Chippaux JP. Snake-bites: appraisal of the global situation. Bull World Health Organ. 1998;76(5):515–24. 9868843 PMC2305789

[4] Warrell DA. Guidelines for the management of snake-bites. Warrell DA, editor. Geneva: World Health Organization; 2010.

[5] Mukherjee AK. Prevention and treatment of the “big four” snakebite in India. The’Big Four’Snakes of India: Venom Composition, Pharmacological Properties and Treatment of Envenomation: Springer; 2021. p. 145–61.

[6] Puzari U, Mukherjee AK. Recent developments in diagnostic tools and bioanalytical methods for analysis of snake venom: A critical review. Anal Chim Acta. 2020;1137:208–24. doi: 10.1016/j.aca.2020.07.054 33153604

[7] Kakati H, Giri S, Patra A, Taye SJ, Agarwalla D, Boruah H, et al. A retrospective analysis of epidemiology, clinical features of envenomation, and in-patient management of snakebites in a model secondary hospital of Assam, North-east India. Toxicon. 2023;230:107175. doi: 10.1016/j.toxicon.2023.107175 37257518

[8] Naik BS. “Dry bite” in venomous snakes: A review. Toxicon. 2017;133:63–7. doi: 10.1016/j.toxicon.2017.04.015 28456535

[9] Puzari U, Fernandes PA, Mukherjee AK. Advances in the Therapeutic Application of Small-Molecule Inhibitors and Repurposed Drugs against Snakebite. J Med Chem. 2021;64(19):13938–79. doi: 10.1021/acs.jmedchem.1c00266 34565143

[10] Williams HF, Layfield HJ, Vallance T, Patel K, Bicknell AB, Trim SA, et al. The Urgent Need to Develop Novel Strategies for the Diagnosis and Treatment of Snakebites. Toxins (Basel). 2019;11(6):363. doi: 10.3390/toxins11060363 31226842 PMC6628419

[11] Warrell DA. Snake bite. Lancet. 2010;375(9708):77–88. doi: 10.1016/S0140-6736(09)61754-2 20109866

[12] Selvanayagam ZE, Gnanavendhan SG, Ganesh KA, Rajagopal D, Rao PV. ELISA for the detection of venoms from four medically important snakes of India. Toxicon. 1999;37(5):757–70. doi: 10.1016/s0041-0101(98)00215-3 10219987

[13] Gao R, Zhang Y, Gopalakrishnakone P. Single-bead-based immunofluorescence assay for snake venom detection. Biotechnol Prog. 2008;24(1):245–9. doi: 10.1021/bp070099e 18179224

[14] Shaikh IK, Dixit PP, Pawade BS, Waykar IG. Development of dot-ELISA for the detection of venoms of major Indian venomous snakes. Toxicon. 2017;139:66–73. doi: 10.1016/j.toxicon.2017.10.007 29024771

[15] Dong L, Eng KH, Quyen L, Gopalakrishnakone P. Optical immunoassay for snake venom detection. Biosens Bioelectron. 2004;19(10):1285–94. 10.1016/j.bios.2003.11.020 15046761

[16] Pawade BS, Salvi NC, Shaikh IK, Waghmare AB, Jadhav ND, Wagh VB, et al. Rapid and selective detection of experimental snake envenomation–Use of gold nanoparticle based lateral flow assay. Toxicon. 2016;119:299–306. 10.1016/j.toxicon.2016.06.023 27377230

[17] Kaul S, Sai Keerthana L, Kumar P, Birader K, Tammineni Y, Rawat D, et al. Cytotoxin antibody-based colourimetric sensor for field-level differential detection of elapid among big four snake venom. PLoS Negl Trop Dis. 2021;15(10):e0009841. doi: 10.1371/journal.pntd.0009841 34634067 PMC8530336

[18] Anand A, Chatterjee B, Dhiman A, Goel R, Khan E, Malhotra A, et al. Complex target SELEX-based identification of DNA aptamers against *Bungarus caeruleus* venom for the detection of envenomation using a paper-based device. Biosensors and Bioelectronics. 2021;193:113523. doi: 10.1234/example.doi 34333364

[19] Suntrarachun S, Pakmanee N, Tirawatnapong T, Chanhome L, Sitprija V. Development of a polymerase chain reaction to distinguish monocellate cobra (Naja khouthia) bites from other common Thai snake species, using both venom extracts and bite-site swabs. Toxicon. 2001;39(7):1087–90. doi: 10.1016/s0041-0101(00)00246-4 11223099

[20] Zhao J, Cui G, Xin M, Tang S. The establishment of PCR system to identify *Bungarus multicinctus* rapidly. Acta Pharm Sin. 2010;45(10):1327–32. doi: DOIifavailable 21348315

[21] Chanda A, Mukherjee AK. Quantitative proteomics to reveal the composition of Southern India spectacled cobra (*Naja naja*) venom and its immunological cross-reactivity towards commercial antivenom. Int J Biol Macromol. 2020;160:224–32. doi: 10.1016/j.ijbiomac.2020.05.106 32439440

[22] Chanda A, Kalita B, Patra A, Senevirathne WDST, Mukherjee AK. Proteomic analysis and antivenomics study of Western India Naja naja venom: correlation between venom composition and clinical manifestations of cobra bite in this region. Expert Rev Proteomics. 2019;16(2):171–84. doi: 10.1080/14789450.2019.1559735 30556786

[23] Patra A, Chanda A, Mukherjee AK. Quantitative proteomic analysis of venom from Southern India common krait (*Bungarus caeruleus*) and identification of poorly immunogenic toxins by immune-profiling against commercial antivenom. Expert Rev Proteomics. 2019;16(5):457–69. doi: 10.1080/14789450.2019.1609945 31002271

[24] Patra A, Kalita B, Chanda A, Mukherjee AK. Proteomics and antivenomics of *Echis carinatus carinatus* venom: Correlation with pharmacological properties and pathophysiology of envenomation. Sci Rep. 2017;7(1):17119. doi: 10.1038/s41598-017-17227-y 29215036 PMC5719401

[25] Kalita B, Patra A, Mukherjee AK. Unraveling the proteome composition and immuno-profiling of Western India Russell’s viper venom for in-depth understanding of its pharmacological properties, clinical manifestations, and effective antivenom treatment. J Proteome Res. 2017;16(2):583–98. doi: 10.1021/acs.jproteome.6b00693 27936776

[26] Kalita B, Singh S, Patra A, Mukherjee AK. Quantitative proteomic analysis and antivenom study revealing that neurotoxic phospholipase A2 enzymes, the major toxin class of Russell’s viper venom from southern India, shows the least immuno-recognition and neutralization by commercial polyvalent antivenom. Int J Biol Macromol. 2018;118(Pt A):375–85. doi: 10.1016/j.ijbiomac.2018.06.083 29924981

[27] Dutta S, Chanda A, Kalita B, Islam T, Patra A, Mukherjee AK. Proteomic analysis to unravel the complex venom proteome of eastern India Naja naja: Correlation of venom composition with its biochemical and pharmacological properties. J Proteomics. 2017;156:29–39. doi: 10.1016/j.jprot.2016.12.018 28062377

[28] Kalita B, Patra A, Das A, Mukherjee AK. Proteomic Analysis and Immuno-Profiling of Eastern India Russell’s Viper (*Daboia russelii*) Venom: Correlation between RVV Composition and Clinical Manifestations Post RV Bite. J Proteome Res. 2018;17(8):2819–33. doi: 10.1021/acs.jproteome.8b00291 29938511

[29] Yap MKK, Tan NH, Sim SM, Fung SY, Tan CH. Pharmacokinetics of Naja sumatrana (equatorial spitting cobra) venom and its major toxins in experimentally envenomed rabbits. PLoS Negl Trop Dis. 2014;8(6):e2890. doi: 10.1371/journal.pntd.0002890 24901441 PMC4046969

[30] Maduwage K, O’Leary M, Isbister G. Diagnosis of snake envenomation using a simple phospholipase A2 assay. Scientific Reports. 2014;4(1):4827. 24777205 10.1038/srep04827 PMC4003729

[31] de Carvalho Oliveira G, Machado CCS, Inácio DK, Silveira Petruci JF da, Silva SG. RGB color sensor for colorimetric determinations: Evaluation and quantitative analysis of colored liquid samples. Talanta. 2022;241:123244. doi: 10.1016/j.talanta.2022.123244 35121545

[32] Solra M, Das S, Rana S. Point-of-care detection of hydroxyurea drug in serum using a supramolecular enzyme mimetic. Sensors and Actuators B: Chemical. 2024;406:135424. doi: 10.1016/j.snb.2024.135424

[33] Aqillah F, Permana M, Eddy D, Firdaus M, Takei T, Rahayu I. Detection and quantification of Cu2+ ion using gold nanoparticles via Smartphone-based digital imaging colorimetry technique. Results in Chemistry. 2024;7:101418.

[34] Albizu G, Bordagaray A, Davila S, Garcia-Arrona R, Ostra M, Vidal M. Analytical control of nickel coating baths by digital image analysis. Microchemical Journal. 2020;154:104600.

[35] Benedetti LP dos S, dos Santos VB, Silva TA, Filho EB, Martins VL, Fatibello-Filho O. A digital image-based method employing a spot-test for quantification of ethanol in drinks. Anal Methods. 2015;7(10):4138–44. doi: 10.1039/c5ay00529a

[36] Puzari U, Khan MR, Mukherjee AK. Development of a gold nanoparticle-based novel diagnostic prototype for in vivo detection of Indian red scorpion (Mesobuthus tamulus) venom. Toxicon X. 2024;23:100203. doi: 10.1016/j.toxcx.2024.100203 39263685 PMC11387954

[37] Kolaskar AS, Tongaonkar PC. A semi-empirical method for prediction of antigenic determinants on protein antigens. FEBS Lett. 1990;276(1–2):172–4. doi: 10.1016/0014-5793(90)80535-q 1702393

[38] Puzari U, Goswami M, Rani K, Patra A, Mukherjee A. Computational and in vitro analyses to identify the anticoagulant regions of Echicetin, a snake venom anticoagulant C-type lectin (snaclec): possibility to develop anticoagulant peptide therapeutics?. J Biomol Struct Dyn. 2023; 1–15. 10.1080/07391102.2023.2191138 36994880

[39] Kakati H, Patra A, Kalita B, Chanda A, Rapole S, Mukherjee AK. A comparison of two different analytical workflows to determine the venom proteome composition of Naja kaouthia from North-East India and immunological profiling of venom against commercial antivenoms. Int J Biol Macromol. 2022;208:275–87. doi: 10.1016/j.ijbiomac.2022.03.095 35331793

[40] Patra A, Banerjee D, Dasgupta S, Mukherjee AK. The in vitro laboratory tests and mass spectrometry-assisted quality assessment of commercial polyvalent antivenom raised against the “Big Four” venomous snakes of India. Toxicon. 2021;192:15–31. doi: 10.1016/j.toxicon.2020.12.015 33417947

[41] Dutta S, Sinha A, Dasgupta S, Mukherjee AK. Binding of a Naja naja venom acidic phospholipase A2 cognate complex to membrane-bound vimentin of rat L6 cells: Implications in cobra venom-induced cytotoxicity. Biochim Biophys Acta Biomembr. 2019;1861(5):958–77. doi: 10.1016/j.bbamem.2019.02.002 30776333

[42] Mukherjee AK, Kalita B, Mackessy SP. A proteomic analysis of Pakistan Daboia russelii russelii venom and assessment of potency of Indian polyvalent and monovalent antivenom. J Proteomics. 2016;144:73–86. doi: 10.1016/j.jprot.2016.06.001 27265321

[43] Das B, Patra A, Puzari U, Deb P, Mukherjee AK. In vitro laboratory analyses of commercial anti-scorpion (Mesobuthus tamulus) antivenoms reveal their quality and safety but the prevalence of a low proportion of venom-specific antibodies. Toxicon. 2022;215:37–48. doi: 10.1016/j.toxicon.2022.06.001 35675849

[44] Frens G. Controlled nucleation for the regulation of the particle size in monodisperse gold suspensions. Nat Phys Sci. 1973;241(105):20–2.

[45] Kimling J, Maier M, Okenve B, Kotaidis V, Ballot H, Plech A. Turkevich method for gold nanoparticle synthesis revisited. J Phys Chem B. 2006;110(32):15700–7. doi: 10.1021/jp061667w 16898714

[46] Herizchi R, Abbasi E, Milani M, Akbarzadeh A. Current methods for synthesis of gold nanoparticles. Artif Cells Nanomed Biotechnol. 2016;44(2):596–602. doi: 10.3109/21691401.2014.971807 25365243

[47] Stuart BH. Infrared spectroscopy: fundamentals and applications. England: John Wiley & Sons; 2004.

[48] Firdaus ML, Alwi W, Trinoveldi F, Rahayu I, Rahmidar L, Warsito K. Determination of Chromium and Iron Using Digital Image-based Colorimetry. Procedia Environmental Sciences. 2014;20:298–304. doi: 10.1016/j.proenv.2014.03.037

[49] Sabitha P, Bammigatti C, Deepanjali S, Suryanarayana BS, Kadhiravan T. Point-of-care infrared thermal imaging for differentiating venomous snakebites from non-venomous and dry bites. PLoS Negl Trop Dis. 2021;15(2):e0008580. doi: 10.1371/journal.pntd.0008580 33600429 PMC7924804

[50] Vaiyapuri R, Williams HF, inventors; ToxiVen Biotech Private Limited, assignee. METHODS, COMPOSITIONS AND KITS FOR VENOM DETECTION. India 2019 16/08/2019.

[51] Lorven B, inventor; LORVEN BIOLOGICALS PVT. LTD, assignee. SNAKE VENOM DETECTION KIT. India 2018 26/07/2018.

[52] Tasoulis T, Isbister GK. A current perspective on snake venom composition and constituent protein families. Arch Toxicol. 2023;97(1):133–53. doi: 10.1007/s00204-022-03420-0 36437303

[53] Ratanabanangkoon K, Tan KY, Pruksaphon K, Klinpayom C, Gutiérrez JM, Quraishi NH, et al. A pan-specific antiserum produced by a novel immunization strategy shows a high spectrum of neutralization against neurotoxic snake venoms. Sci Rep. 2020;10(1):11261. doi: 10.1038/s41598-020-66657-8 32647261 PMC7347863

[54] Ainsworth S, Slagboom J, Alomran N, Pla D, Alhamdi Y, King S. The paraspecific neutralisation of snake venom induced coagulopathy by antivenoms. Comm Biol. 2018;1(1):34. 10.1038/s42003-018-0039-1 PMC6123674 30271920

[55] Tan CH, Liew JL, Tan NH, Ismail AK, Maharani T, Khomvilai S, et al. Cross reactivity and lethality neutralization of venoms of Indonesian Trimeresurus complex species by Thai Green Pit Viper Antivenom. Toxicon. 2017;140:32–7. doi: 10.1016/j.toxicon.2017.10.014 29051104

[56] Ramana LN, Mathapati SS, Salvi N, Khadilkar MV, Malhotra A, Santra V, et al. A paper microfluidic device based colorimetric sensor for the detection and discrimination of elapid versus viper envenomation. Analyst. 2022;147(4):685–94. doi: 10.1039/d1an01698a 35072182

[57] Trier NH, Hansen PR, Houen G. Production and characterization of peptide antibodies. Methods. 2012;56(2):136–44. doi: 10.1016/j.ymeth.2011.12.001 22178691

[58] Lee B-S, Huang J-S, Jayathilaka LP, Lee J, Gupta S. Antibody production with synthetic peptides. In: Schwartzbach S, Skalli O, Schikorski T, editor. Methods in Molecular Biology 1474. NY: Humana Press, New York; 2016. p. 25–47. 10.1007/978-1-4939-6352-2_2 27515072

[59] Trier N, Hansen P, Houen G. Peptides, Antibodies, Peptide Antibodies and More. Int J Mol Sci. 2019;20(24):6289. doi: 10.3390/ijms20246289 31847088 PMC6941022

[60] Madhubala D, Patra A, Islam T, Saikia K, Khan MR, Ahmed SA, et al. Snake venom nerve growth factor-inspired designing of novel peptide therapeutics for the prevention of paraquat-induced apoptosis, neurodegeneration, and alteration of metabolic pathway genes in the rat pheochromocytoma PC-12 cell. Free Radic Biol Med. 2023;197:23–45. 36669545 10.1016/j.freeradbiomed.2023.01.019

[61] Schaaper WM, Lankhof H, Puijk WC, Meloen RH. Manipulation of antipeptide immune response by varying the coupling of the peptide with the carrier protein. Mol Immunol. 1989;26(1):81–5. doi: 10.1016/0161-5890(89)90023-0 2538727

[62] Houen G, Olsen DT, Hansen PR, Petersen KB, Barkholt V. Preparation of bioconjugates by solid-phase conjugation to ion exchange matrix-adsorbed carrier proteins. Bioconjug Chem. 2003;14(1):75–9. doi: 10.1021/bc025622j 12526695

[63] Deka A, Bhatia S, Santra V, Bharti OK, Lalremsanga HT, Martin G, et al. Multilevel Comparison of Indian *Naja* Venoms and Their Cross-Reactivity with Indian Polyvalent Antivenoms. Toxins (Basel). 2023;15(4):258. doi: 10.3390/toxins15040258 37104196 PMC10142961

[64] Senji Laxme RR, Khochare S, de Souza HF, Ahuja B, Suranse V, Martin G, et al. Beyond the “big four”: Venom profiling of the medically important yet neglected Indian snakes reveals disturbing antivenom deficiencies. PLoS Negl Trop Dis. 2019;13(12):e0007899. doi: 10.1371/journal.pntd.0007899 31805055 PMC6894822

[65] Domínguez-Oliva A, Hernández-Ávalos I, Martínez-Burnes J, Olmos-Hernández A, Verduzco-Mendoza A, Mota-Rojas D. The Importance of Animal Models in Biomedical Research: Current Insights and Applications. Animals (Basel). 2023;13(7):1223. doi: 10.3390/ani13071223 37048478 PMC10093480

[66] Huang G, Ashton C, Kumbhani DS, Ying Q-L. Genetic manipulations in the rat: progress and prospects. Curr Opin Nephrol Hypertens. 2011;20(4):391–9. doi: 10.1097/MNH.0b013e328347768a 21546835 PMC3857098

[67] Hashway SA, Wilding LA. Translational potential of rats in research. In: Suckow MA, Hankenson FC, Wilson RP, Foley PL, editors. The laboratory rat. Elsevier; 2020. p. 77–88.

[68] Anai K, Sugiki M, Yoshida E, Maruyama M. Neutralization of a snake venom hemorrhagic metalloproteinase prevents coagulopathy after subcutaneous injection of *Bothrops jararaca* venom in rats. Toxicon. 2002;40(1):63–8. doi: 10.1016/s0041-0101(01)00189-1 11602280

[69] Gutiérrez JM, Calvete JJ, Habib AG, Harrison RA, Williams DJ, Warrell DA. Snakebite envenoming. Nat Rev Dis Primers. 2017;3:17079. doi: 10.1038/nrdp.2017.79 28980622

[70] Paniagua D, Vergara I, Boyer L, Alagón A. Role of lymphatic system on snake venom absorption. In: Gopalakrishnakone HIP, Vogel C-W, Mukherjee AK, Rahmy TR, editor. Snake Venoms. Dordrecht. Springer: Springer Netherlands; 2017. p. 453–74.

[71] Hung D-Z, Liau M-Y, Lin-Shiau S-Y. The clinical significance of venom detection in patients of cobra snakebite. Toxicon. 2003;41(4):409–15. doi: 10.1016/s0041-0101(02)00336-7 12657310

[72] Sanhajariya S, Duffull SB, Isbister GK. Pharmacokinetics of Snake Venom. Toxins (Basel). 2018;10(2):73. doi: 10.3390/toxins10020073 29414889 PMC5848174

[73] Guo MP, Wang QC, Liu GF. Pharmacokinetics of cytotoxin from Chinese cobra (Naja naja atra) venom. Toxicon. 1993;31(3):339–43. doi: 10.1016/0041-0101(93)90151-8 8470137

[74] Choowongkomon K, Chaisakul J, Seetaha S, Vasaruchapong T, Hodgson WC, Rasri N, et al. Development of a Biosensor to Detect Venom of Malayan Krait (Bungarus candidus). Toxins (Basel). 2024;16(1):56. doi: 10.3390/toxins16010056 38276532 PMC10820552

[75] Ngernpimai S, Srijampa S, Thongmee P, Teerasong S, Puangmali T, Maleewong W, et al. Insight into the Covalently Oriented Immobilization of Antibodies on Gold Nanoparticle Probes to Improve Sensitivity in the Colorimetric Detection of Listeria monocytogenes. Bioconjug Chem. 2022;33(11):2103–12. doi: 10.1021/acs.bioconjchem.2c00261 36273419

[76] Bastos-Soares EA, da Silva Morais MS, Funes-Huacca M, Sousa RMO, Brilhante-Da-Silva N, Roberto SA, et al. Single-Domain Antibody-Gold Nanoparticle Bioconjugates as Immunosensors for the Detection of Hantaviruses. Mol Diag Ther. 2024;1–16. 10.1007/s40291-024-00713-1 38796660

[77] Potluri V, Kathiresan PS, Kandula H, Thirumalaraju P, Kanakasabapathy MK, Kota Sai Pavan S, et al. An inexpensive smartphone-based device for point-of-care ovulation testing. Lab Chip. 2018;19(1):59–67. doi: 10.1039/c8lc00792f 30534677 PMC6321627

[78] Tong H, Cao C, You M, Han S, Liu Z, Xiao Y, et al. Artificial intelligence-assisted colorimetric lateral flow immunoassay for sensitive and quantitative detection of COVID-19 neutralizing antibody. Biosens Bioelectron. 2022;213:114449. doi: 10.1016/j.bios.2022.114449 35696869 PMC9174064

[79] Slagboom J, Kool J, Harrison RA, Casewell NR. Haemotoxic snake venoms: their functional activity, impact on snakebite victims and pharmaceutical promise. Br J Haematol. 2017;177(6):947–59. doi: 10.1111/bjh.14591 28233897 PMC5484289

[80] Gamulin E, Mateljak Lukačević S, Halassy B, Kurtović T. Snake Antivenoms-Toward Better Understanding of the Administration Route. Toxins (Basel). 2023;15(6):398. doi: 10.3390/toxins15060398 37368699 PMC10302821

[81] Mohapatra B, Warrell DA, Suraweera W, Bhatia P, Dhingra N, Jotkar RM, et al. Snakebite mortality in India: a nationally representative mortality survey. PLoS Negl Trop Dis. 2011;5(4):e1018. doi: 10.1371/journal.pntd.0001018 21532748 PMC3075236

[82] Pucca MB, Knudsen C, S Oliveira I, Rimbault C, A Cerni F, Wen FH, et al. Current Knowledge on Snake Dry Bites. Toxins (Basel). 2020;12(11):668. doi: 10.3390/toxins12110668 33105644 PMC7690386

[83] Organization WH. Guidelines for the clinical management of snake bites in the South-East Asia Region. New Delhi WHO South East Asia Regional Office, 2005.

