# Supplementary material for: Diagnosis of Indian Big Four and monocled Cobra snakebites in envenomed plasma using smartphone-based digital imaging colourimetry method
Source: PLoS Negl Trop Dis. 2025 Mar 14;19(3):e0012913. doi: 10.1371/journal.pntd.0012913 (PMC11936222; doi:10.1371/journal.pntd.0012913)
Supplement: S1 Text — Methodologies for: KLH conjugation of the CPs, Raising and purifying custom peptide-specific antibodies by immunising rabbits with KLH-conjugated CPs and Synthesis of AuNP-FPAb conjugates. (DOCX) [file pntd.0012913.s001.docx]

**Diagnosis of Indian Big Four and monocled Cobra snakebites in envenomed plasma using smartphone-based digital imaging colourimetry method**

**Upasana Puzari^1^, Mojibur R. Khan^2,3^, Ashis K. Mukherjee*^1,2,3^**

^1^Microbial Biotechnology and Protein Research Laboratory, Department of Molecular Biology and Biotechnology, School of Sciences, Tezpur University, Tezpur- 784028, Assam, India

^2^Division of Life Sciences, Institute of Advanced Study in Science and Technology, Vigyan Path Garchuk, Paschim Boragaon, Guwahati 781035, Assam, India

^3^Academy of Science and Innovative Research (AcSIR), Ghaziabad, India

***Corresponding author:** Dr. A. K. Mukherjee, Institute of Advanced Study in Science

and Technology, Guwahati- 781035, Assam, India.

**ORCID iD:** 0000-0001-9869-858X.

**Email address:** akm@tezu.ernet.in; ashmukh@yahoo.co.uk

**Methodologies:**

**KLH conjugation of the CPs**

Since the CPs alone may be too small to elicit a sufficient immune response, each of the CPs was conjugated with the carrier protein Keyhole Limpet Hemocyanin (KLH) using the method described by [1]. Briefly, 10 mg/mL KLH solution was prepared in 10 mM phosphate buffer, pH 7.0 and 70 µL m-maleimidobenzoyl-N-hydroxysuccinimide ester (MBS) dissolved in 200 µL dimethyl formamide (DMF) was added to it. Free MBS-DMF crosslinkers were removed by Sephadex G25 size exclusion chromatography. The column was equilibrated in 50 mM phosphate buffer (pH 7.0), and the KLH reaction mixture was loaded onto the column using the same buffer. The void volume that was eluted first contained KLH with a molecular weight of ~400 kDa. The CPs were dissolved in 100 μL DMF and mixed rapidly with the purified KLH/MBS. The CP-KLH mixture was vortexed, pH was adjusted to 7.0-7.2 using 2N NaOH and stirred overnight at 4°C to allow the conjugation of CPs to KLH. The next day, the solution of KLH conjugated CP was lyophilised, and 0.1 M ammonium bicarbonate was added. The lyophilised KLH-conjugated CPs were outsourced to raise toxin-specific polyclonal antibodies in rabbits.

**Raising and purifying custom peptide-specific antibodies by immunising rabbits with KLH-conjugated CPs**

Briefly, 2 mL of blood was drawn as pre-immune serum from the white New Zealand rabbits. After 24 h, primary immunisation was performed by injecting ~200 μg of KLH-conjugated CPs subcutaneously in Freund's complete adjuvant. The rabbits were then administered the first booster dose of 100 μg KLH-conjugated peptides in Freund's incomplete adjuvant 15 days later. The rabbits were administered a second booster dose (in Freund's incomplete adjuvant) on the 10^th^ day post-first dose. On the subsequent 10^th^ day, serum was separated from an ear vein test bleed, and the antibody titre was checked with ELISA. The step was repeated to administer a third booster dose in Freund's incomplete adjuvant, and a test bleed was done to determine the antibody titre.

The ELISA was performed by coating the microtiter wells with 100 μL solution of 2μg/mL non-conjugated peptide in PBS (200 ng/well) and left overnight at 4°C. The next day, the wells were blocked at room temperature with 250 μL of 1% BSA in PBS for 1h. After incubation, the wells were washed thrice with wash buffer (PBS with 0.05% Tween 20). After incubating the diluted serum samples (100 μL) for 30 min at room temperature, the wells were washed thrice with wash buffer again. Then, the wells were incubated with 100 μL of Protein A-HRP (Zymed, USA) diluted to 1/60,000 for 30 min at room temperature. The wells were then incubated in the dark with 100 μL of TMB/H_2_O_2_ substrate solution for 30 min at room temperature. Lastly, the reaction was stopped by adding 50 μL of 2M H_2_SO_4_, and absorbance was recorded at 450 nm (primary wavelength) and 630 nm (reference wavelength) against the reagent blank. The polyclonal antibodies were purified from the collected antisera using affinity chromatography described in our previous protocol (Puzari et al., 2024) [3].

The immune reactivity of the PAbs against their respective CPs (against which they were raised) was assessed by dot blot analysis [2, 3]. For dot blot analysis, the polyvinylidene fluoride (PVDF) membrane was activated with 100% methanol and equilibrated with 1X TBS with 0.05% tween-20 (TBS-T). Then, 1 µL of KLH-conjugated CPs (2 µg) were spotted onto activated membrane and air-dried. Non-specific binding sites on the membranes were blocked by 5% fat-free skimmed milk and incubated with gentle shaking for 1 h at room temperature. The membranes were washed thrice with TBS-T and incubated with the PAbs (1 µg/µL) at a dilution of 1:1000. The PAbs were detected by anti-rabbit HRP-conjugated secondary antibodies at 1:2000 dilutions. The blots were developed using ECL substrate (Cat no. 1705060, Bio-Rad) using the ChemiDoc imaging system, with Image Lab software (Bio-Rad, USA) and ImageJ software (https://imagej.nih.gov/ij/) used to measure the intensity of the dots. BSA was used as a negative control in the analysis.

**Synthesis of AuNP-FPAb conjugates**

To begin with, citrate-stabilised AuNPs were ligand exchanged with thiolated 11-mercaptoundecanoic acid (MUA) to generate AuNP-MUA. To do this 10 mM MUA was added to AuNPs in a ratio of 1:5 (MUA/AuNP, v/v) and incubated for 24 h under stirring conditions at 100 rpm, at 35°C. The AuNP-MUA was washed thrice by centrifugation (10000 rpm, 25 min) and then suspended in ultrapure water. Bioconjugation of FPAbs to the AuNP-MUAs was performed using the EDC/NHS [EDC:1-Ethyl-3-(3-dimethylaminopropyl) carbodiimide; NHS: N-Hydroxysuccinimide] coupling chemistry. Briefly, 2mL of AuNP-MUAs were incubated with 200 μL of 50 μM EDC under stirring conditions for 30 min at 150 rpm. The reaction mixture was further incubated with 200 μL of 75 μM NHS under stirring for 30 min more. Successively, the reaction mixture was incubated with 20 μg of FPAb (2 µg/µL) for 30 min before cooling it at 4°C overnight. The pellet obtained after centrifuging the reaction mixture (10000 rpm, 20 min) was suspended in 100 μL of phosphate buffer (10 mM, pH 7.4) after adding 1% (w/v) BSA to functionalise the non-antibody coated areas, forming the AuNP-FPAb conjugate.

**References:**

1. Van Regenmortel MH. Synthetic polypeptides as antigens. In: Burdon R, and Knippenberg, P., editor. 19. Amsterdam, Netherlands: Elsevier; 1988.

2. Puzari U, Goswami M, Rani K, Patra A, Mukherjee AK. Computational and in vitro analyses to identify the anticoagulant regions of Echicetin, a snake venom anticoagulant C-type lectin (snaclec): possibility to develop anticoagulant peptide therapeutics? J Biomol Struct Dyn. 2023:1-15.

3. Puzari U, Khan MR, Mukherjee AK. Development of a gold nanoparticle-based novel diagnostic prototype for *in vivo* detection of Indian red scorpion (*Mesobuthus tamulus*) venom. Toxicon: X. 2024:100203.
