# Supplementary figures and images for: Diagnosis of Indian Big Four and monocled Cobra snakebites in envenomed plasma using smartphone-based digital imaging colourimetry method
Source: PLoS Negl Trop Dis. 2025 Mar 14;19(3):e0012913. doi: 10.1371/journal.pntd.0012913 (PMC11936222; doi:10.1371/journal.pntd.0012913)

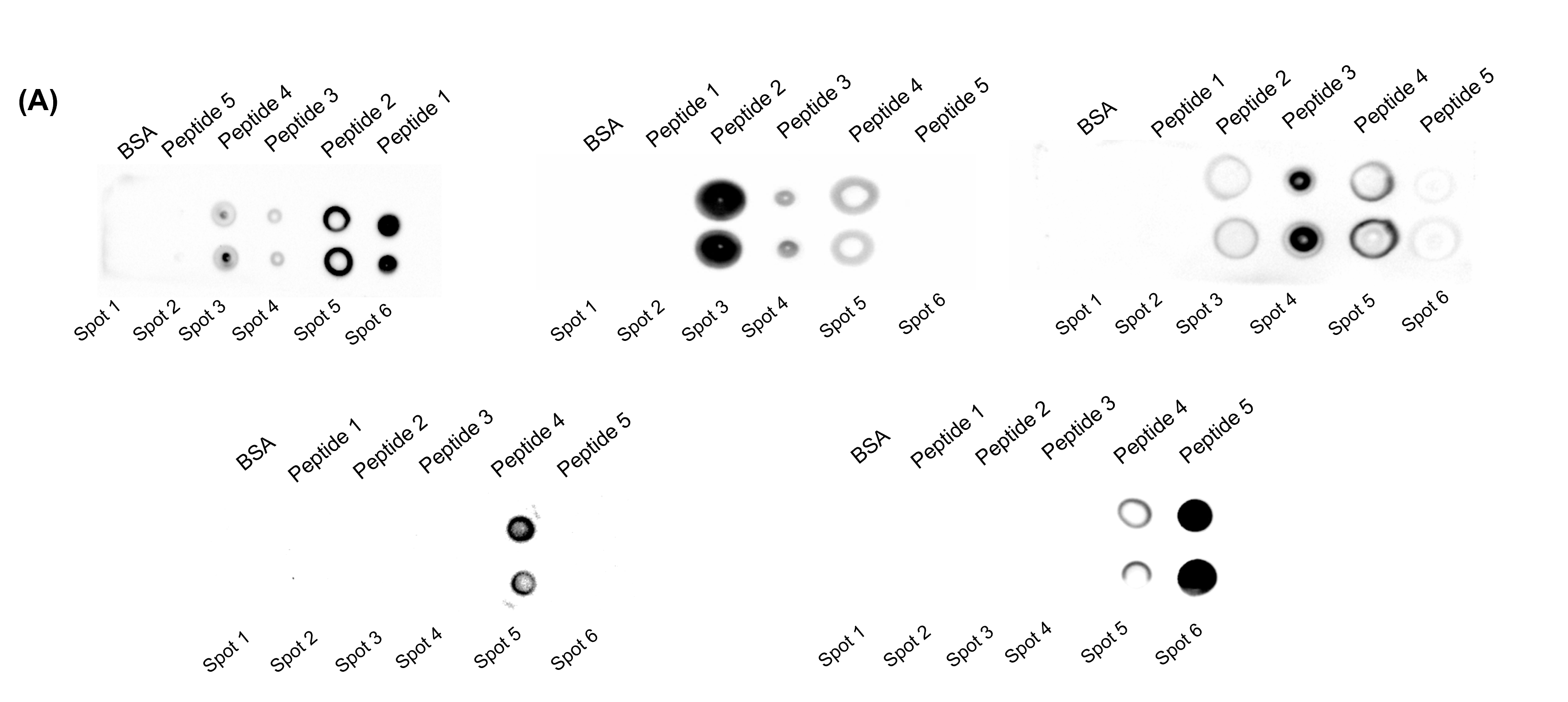

Supplement: S1 Fig — (A) Dot blot assay to determine the immune-recognition of individual PAbs towards the CPs. Dot blot intensities of immune recognition demonstrated by the individual PAbs towards the CPs. (B) PAb 1 demonstrated cross-reactivity towards CP2-5. Significance of difference in recognition of PAb 1 towards CP2, CP3, CP4 and CP5 compared to CP1, *p<0.05. (C) PAb 2 demonstrated some cross-reactivity towards CP3 and CP4. Significance of difference in recognition of PAb 2 towards CP3 and CP4 compared to CP2, ɣp<0.05. (D) PAb 3 demonstrated cross-reactivity towards CP2, CP4 and CP5. Significance of difference in recognition of PAb 3 towards CP2, CP4 and CP5 compared to CP3, ωp<0.05. (E) PAb 4 demonstrated specificity towards CP4. (F) PAb 5 demonstrated cross-reactivity towards CP4. Significance of difference in recognition of PAb 5 towards CP4 compared to CP5, ψp<0.05. Error bars indicate mean ± SD (n=3). (TIFF) [file pntd.0012913.s002.tiff]

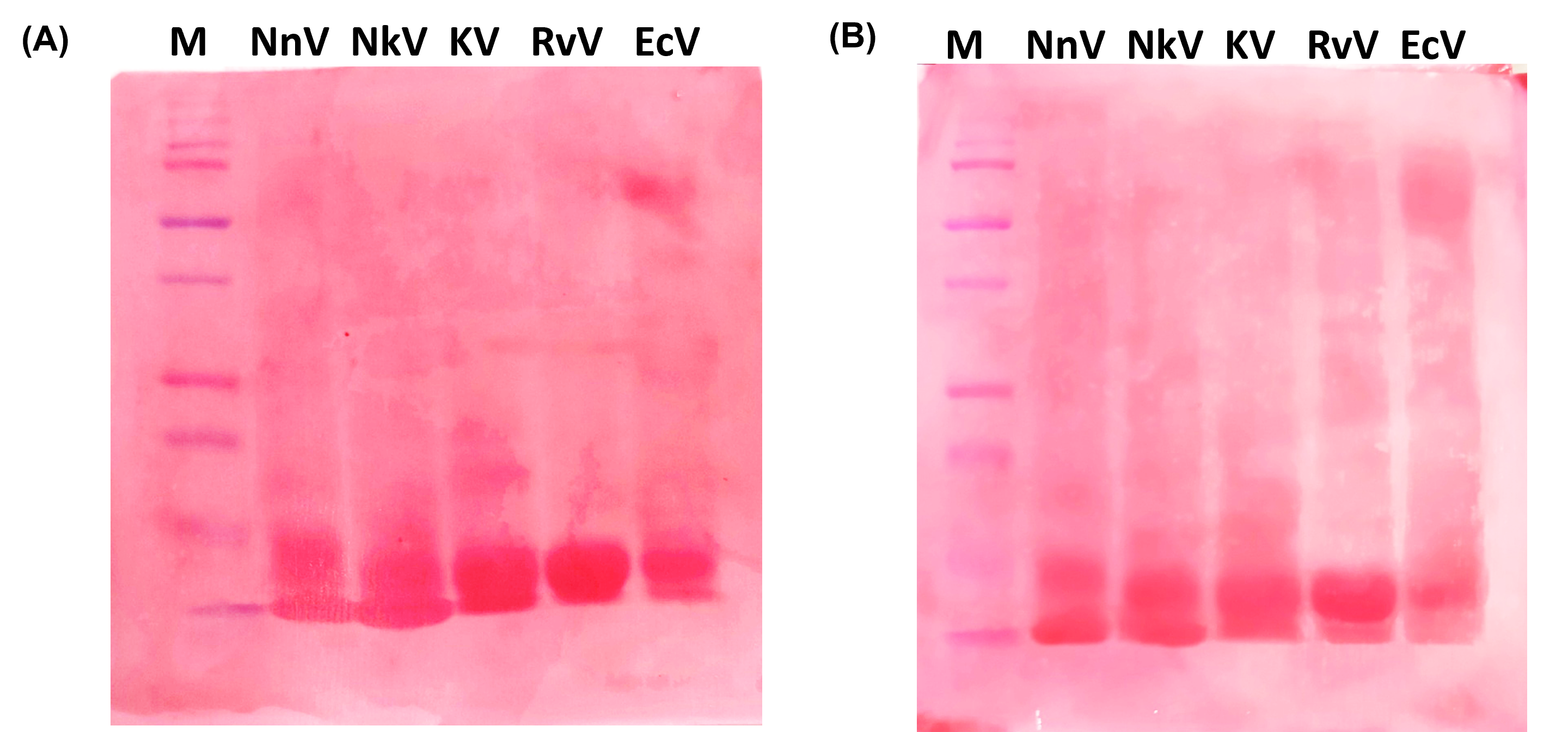

Supplement: S2 Fig — (A, B) Ponceau-S red stained blots indicating snake venom protein transfer. (TIF) [file pntd.0012913.s003.tif]

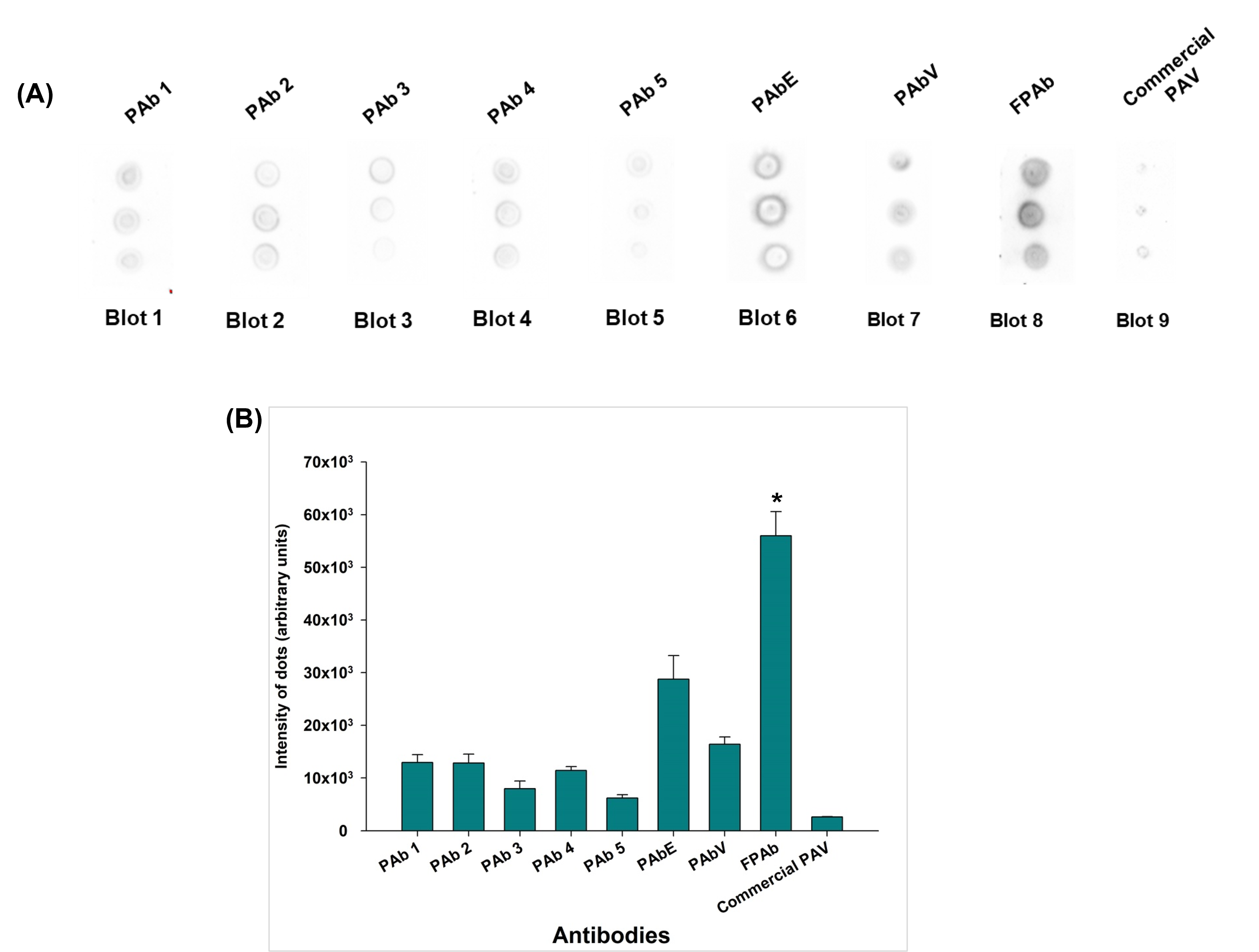

Supplement: S3 Fig — (A) Dot blot assay to determine immune-recognition of PAbs (individual PAb 1, 2, 3, 4, 5, PAbE, PAbV and FPAb) using anti-rabbit IgG-HRP and commercial PAV using anti-horse IgG-HRP; (B) Dot intensities of the immune-recognition demonstrated by the secondary antibodies as stated in (A). Significance of difference immune-recognition of FPAb by anti-rabbit IgG-HRP with respect to immune recognition of individual PAbs, PAbE, PAbV by anti-rabbit IgG-HRP and commercial PAV by anti-horse IgG-HRP, *p<0.05. Error bars indicate mean ± SD (n=3). (TIF) [file pntd.0012913.s004.tif]

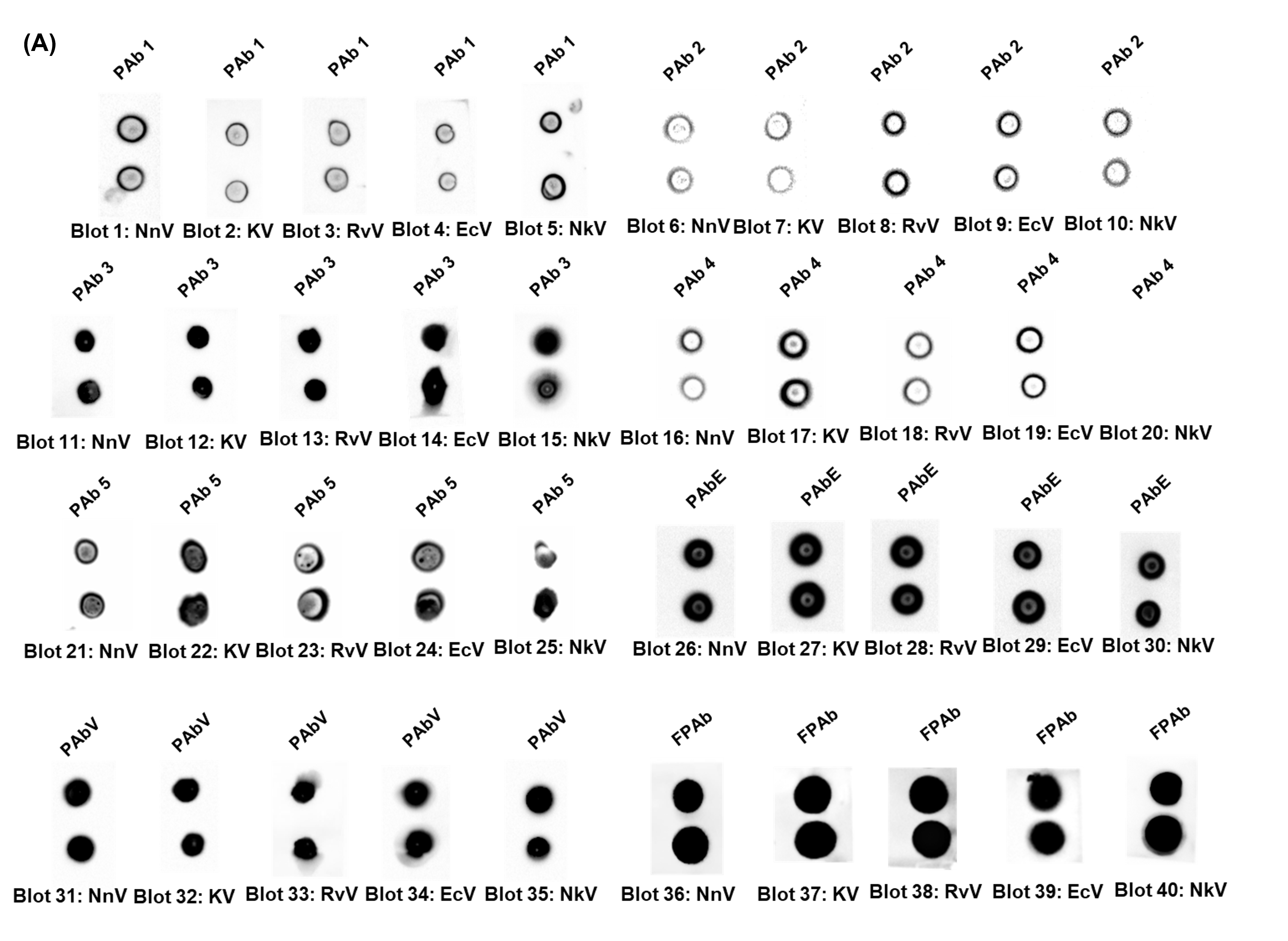

Supplement: S4 Fig — (A) Dot blot assay to determine immune-recognition of PAbs (individual PAb 1, 2, 3, 4, 5, PAbE, PAbV and FPAb) towards NnV, KV, NkV, RvV and EcV (1 pg/μL), (B) Dot intensities of the immune-recognition of NnV, KV, NkV, RvV and EcV by individual PAbs, PAbE, PAbV and FPAb. Significance of difference in immune-recognition of NnV, KV, NkV, RvV and EcV by PAb 1,2,3,4,5, PAbE and PAbV compared to immune-recognition by FPAb *p<0.05. Error bars indicate mean ± SD (n=3). (TIFF) [file pntd.0012913.s005.tiff]

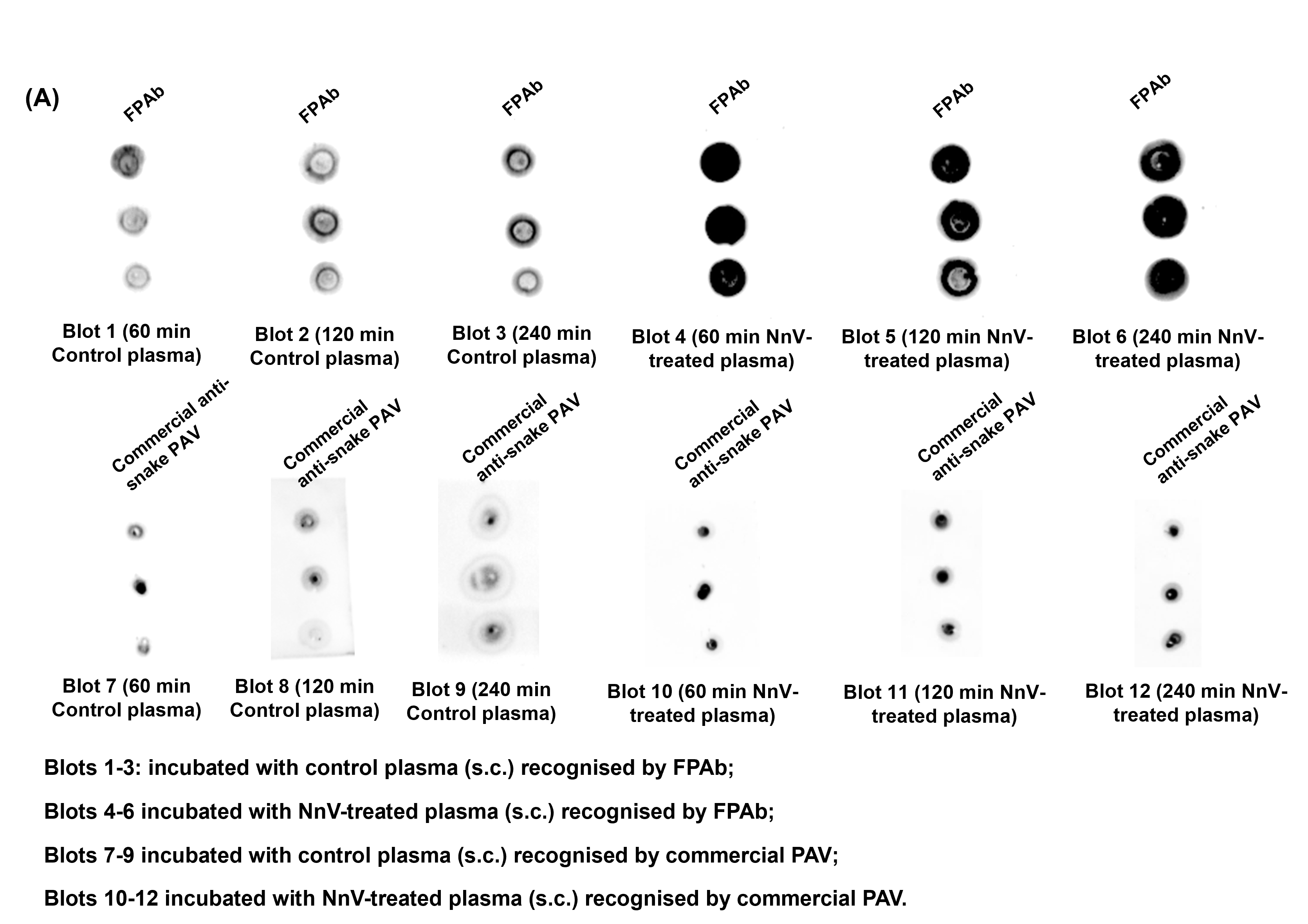

Supplement: S5 Fig — (A) Dot blot assay to determine immune-recognition of NnV in the plasma of the group 1 and 2 rats by FPAb and commercial PAV when the blood was collected at 60 min, 120 min, and 240 min post-injection (s.c.). Blots 1-3 incubated with control plasma (s.c.) collected after 60 min, 120 min, and 240 min recognised by FPAb; Blots 4-6 incubated with NnV -treated plasma (s.c.) collected after 60 min, 120 min, and 240 min recognised by FPAb; Blots 7-9 incubated with control plasma (s.c.) collected after 60 min, 120 min, and 240 min recognised by commercial PAV; Blots 10-12 incubated with NnV -treated plasma (s.c.) collected after 60 min, 120 min, and 240 min recognised by commercial PAV. (B) Dot blot assay to determine immune-recognition of KV in the plasma of the group 1 and 3 rats by FPAb and commercial PAV when the blood was collected at 60 min, 120 min, and 240 min post-injection (s.c.). Blots 1-3 incubated with control plasma (s.c.) collected after 60 min, 120 min, and 240 min recognised by FPAb; Blots 4-6 incubated with KV-treated plasma (s.c.) collected after 60 min, 120 min, and 240 min recognised by FPAb; Blots 7-9 incubated with control plasma (s.c.) collected after 60 min, 120 min, and 240 min recognised by commercial PAV; Blots 10-12 incubated with KV-treated plasma (s.c.) collected after 60 min, 120 min, and 240 min recognised by commercial PAV. (C) Dot blot assay to determine immune-recognition of NkV in the plasma of the group 1 and 6 rats by FPAb and commercial PAV when the blood was collected at 60 min, 120 min, and 240 min post-injection (s.c.). Blots 1-3 incubated with control plasma (s.c.) collected after 60 min, 120 min, and 240 min recognised by FPAb; Blots 4-6 incubated with NkV-treated plasma (s.c.) collected after 60 min, 120 min, and 240 min recognised by FPAb; Blots 7-9 incubated with control plasma (s.c.) collected after 60 min, 120 min, and 240 min recognised by commercial PAV; Blots 10-12 incubated with NkV-treated plasma (s.c.) collected after [file pntd.0012913.s006.tiff]

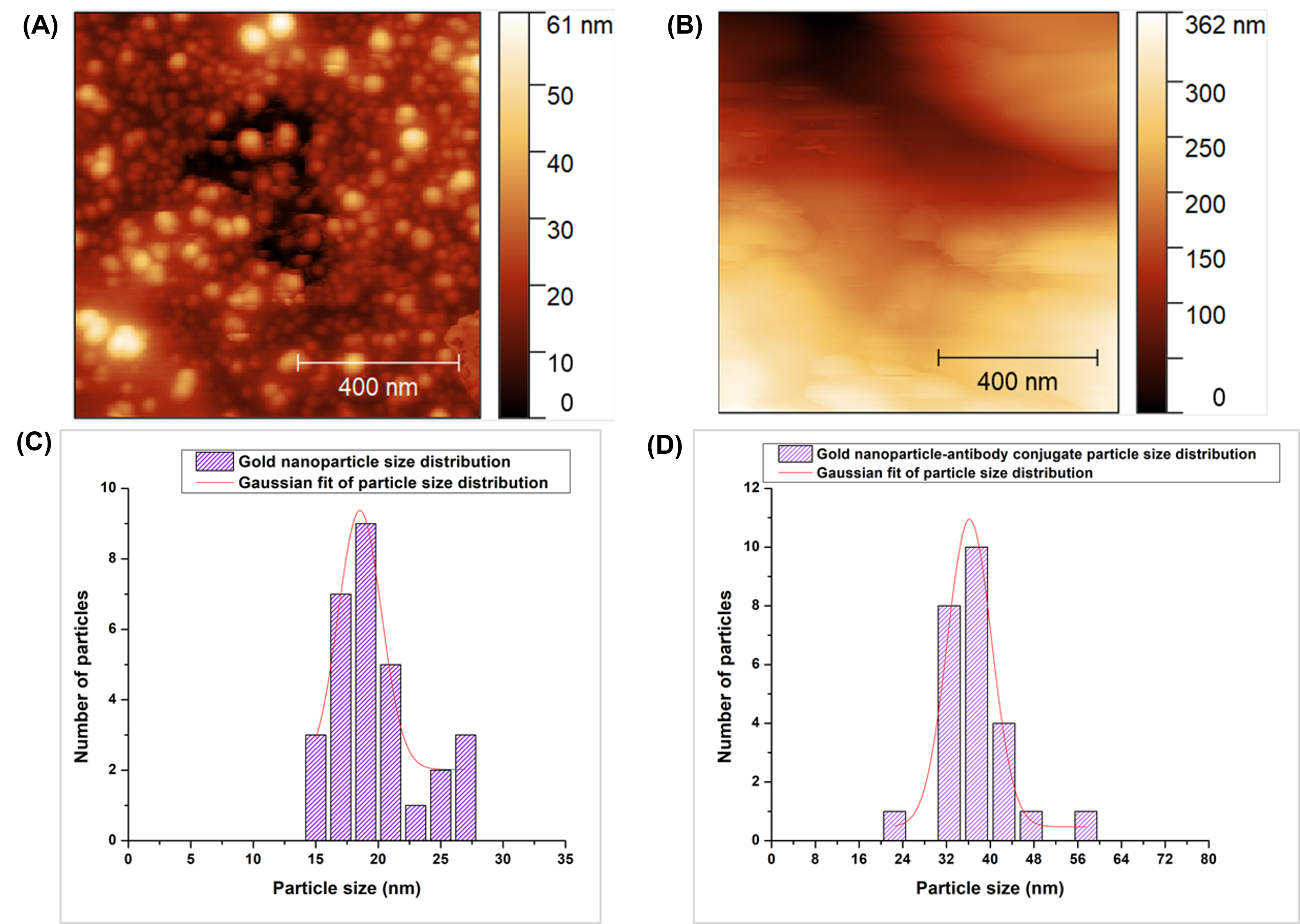

Supplement: S6 Fig — (A) UV-Vis spectra depicting AuNP and AuNP-FPAb conjugate. The absorbance is the mean of values obtained in triplicates; (B) FTIR spectra of AuNP and AuNP-FPAb conjugate; (C) Zeta potential of AuNP and AuNP-FPAb conjugate. (TIF) [file pntd.0012913.s007.tif]

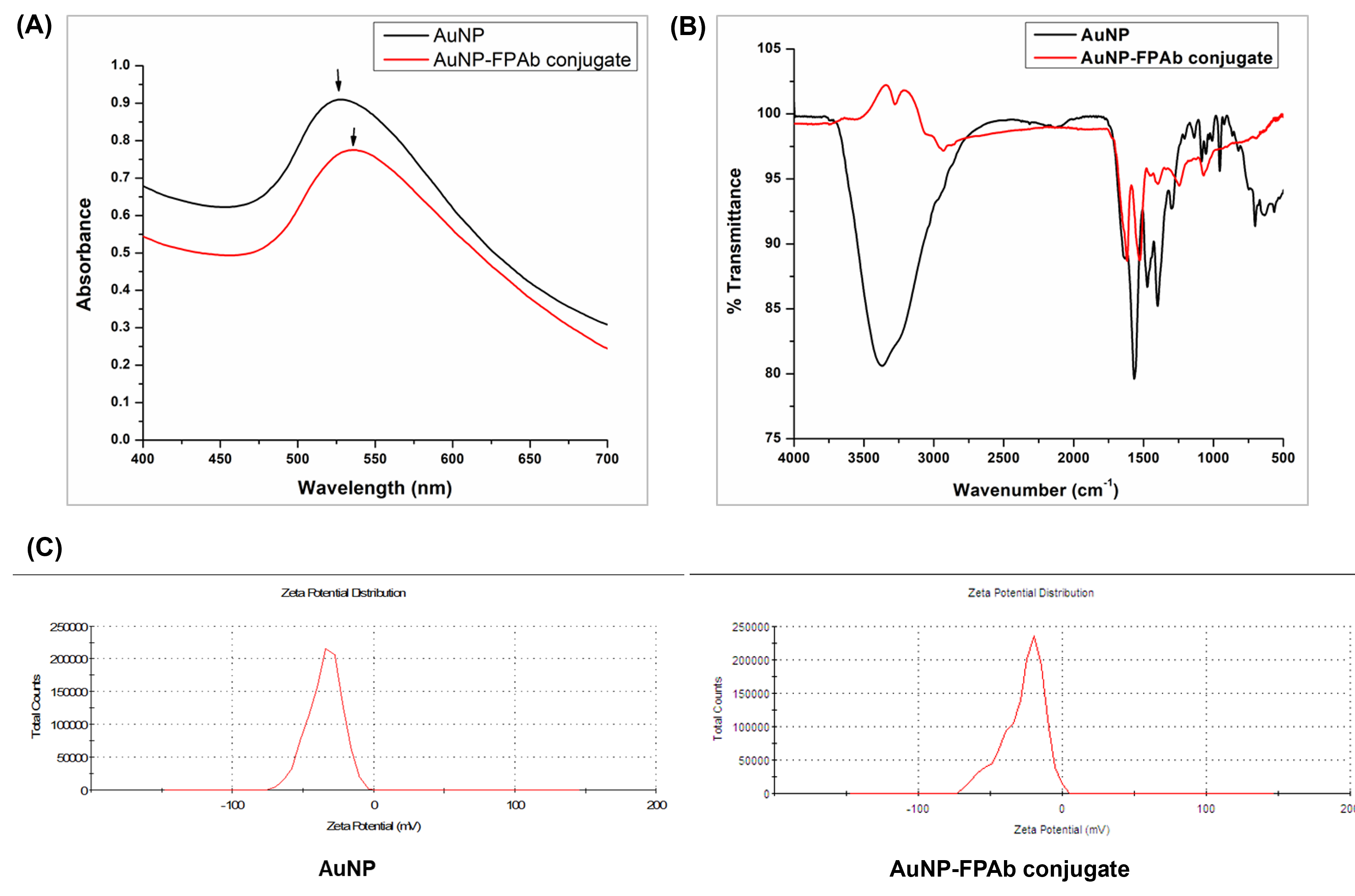

Supplement: S7 Fig — TEM images of (A) AuNP and (B) AuNP-FPAb conjugate particle at 20 nm magnification; Histogram depicting Particle size distribution of (C) AuNP and (D) AuNP- FPAb conjugate particle in TEM images, with Gaussian function, fit using Originpro 8.5. (TIF) [file pntd.0012913.s008.tif]

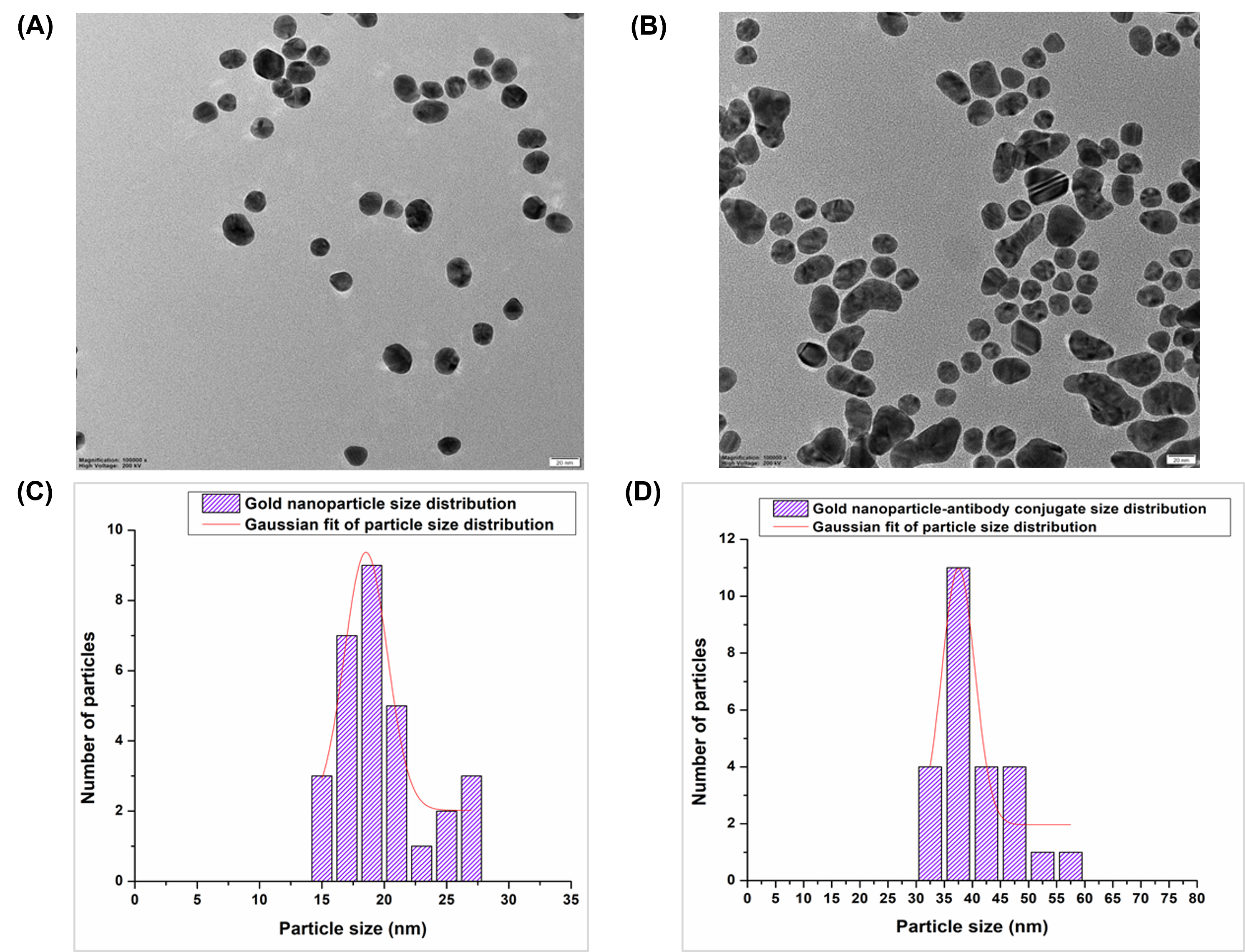

Supplement: S8 Fig — Topographic 2D AFM images with scanned area 1000 x 1000 nm of (A) AuNP, (B) AuNP-FPAb conjugate; Histogram of height distribution of (C) AuNP, (D) AuNP-FPAb conjugate, from the topographic 2D AFM images with scanned area 1000 x 1000 nm. (TIF) [file pntd.0012913.s009.tif]

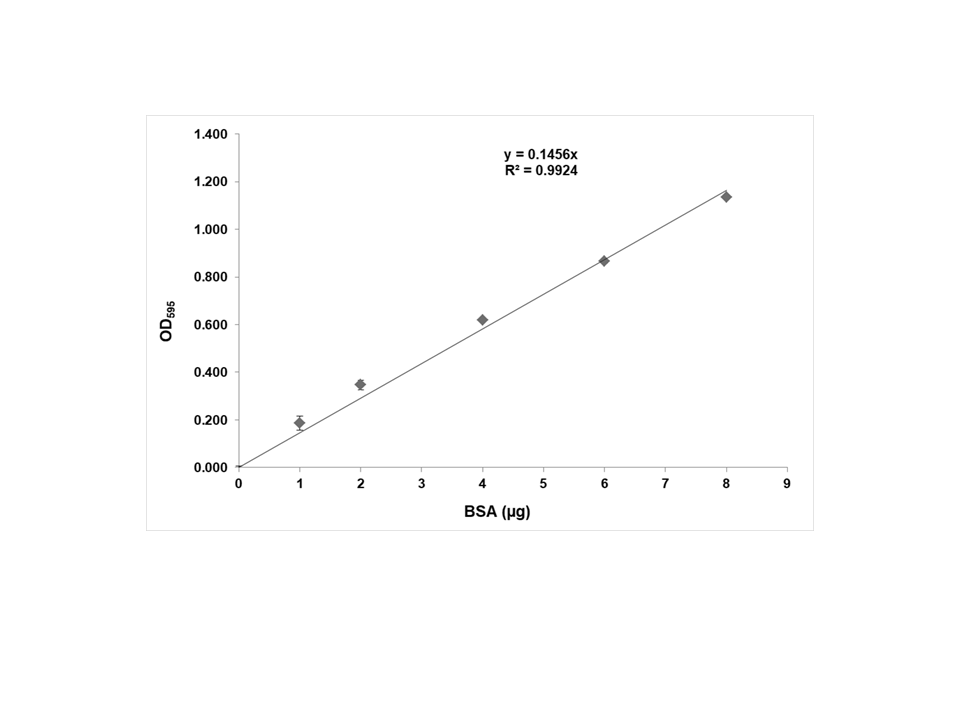

Supplement: S9 Fig — Error bars indicate mean ± SD (n=3). (TIF) [file pntd.0012913.s010.tif]

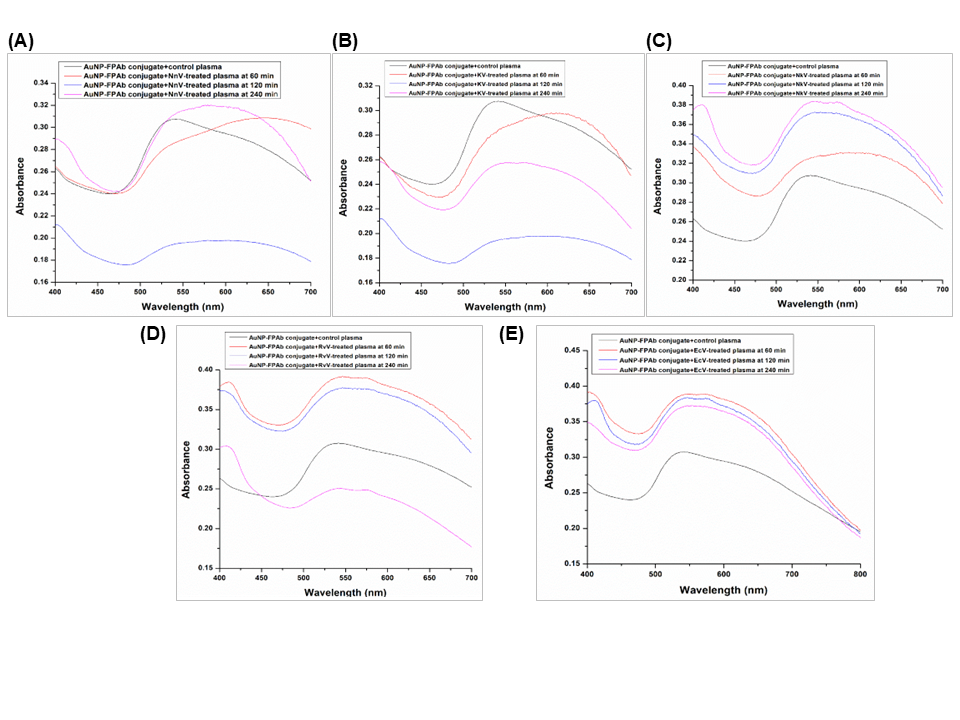

Supplement: S10 Fig — (A) Absorbance spectra of the AuNP-FPAb conjugate in the presence of control (untreated, group 1 rats) and NnV-treated plasma collected at 60 min, 120 min and 240 min post-injection (group 2 rats). The absorption maximum (λmax) for Control plasma was at 537 nm. On interacting with the envenomed plasma, the λmax shifted to 630 nm, 602 nm and 580 nm for NnV-treated plasma collected at 60 min, 120 min and 240 min, respectively.; (B) Absorbance spectra of the AuNP-FPAb conjugate in the presence of control (untreated, group 1 rats) and KV-treated plasma collected at 60 min, 120 min and 240 min post-injection (group 3 rats). The absorption maximum (λmax) for Control plasma was at 537 nm. On interacting with the envenomed plasma, the λmax shifted to 610 nm, 589 nm and 555 nm for KV-treated plasma collected at 60 min, 120 min and 240 min, respectively.; (C) Absorbance spectra of the AuNP-FPAb conjugate in the presence of control (untreated, group 1 rats) and NkV-treated plasma collected at 60 min, 120 min and 240 min post-injection (group 6 rats). The absorption maximum (λmax) for Control plasma was at 537 nm. On interacting with the envenomed plasma, the λmax shifted to 604 nm, 548 nm and 546 nm for NkV-treated plasma collected at 60 min, 120 min and 240 min, respectively.; (D) Absorbance spectra of the AuNP-FPAb conjugate in the presence of control (untreated, group 1 rats) and RvV-treated plasma collected at 60 min, 120 min and 240 min post-injection (group 4 rats). The absorption maximum (λmax) for Control plasma was at 537 nm. On interacting with the envenomed plasma, the λmax shifted to 548 nm, 547 nm and 543 nm for RvV-treated plasma collected at 60 min, 120 min and 240 min, respectively.; (E) Absorbance spectra of the AuNP-FPAb conjugate in the presence of control (untreated, group 1 rats) and EcV-treated plasma collected at 60 min, 120 min and 240 min post-injection (group 5 rats). The absorption maximum (λmax) for Control plasma was at 537 nm. On interacting with [file pntd.0012913.s011.tif]

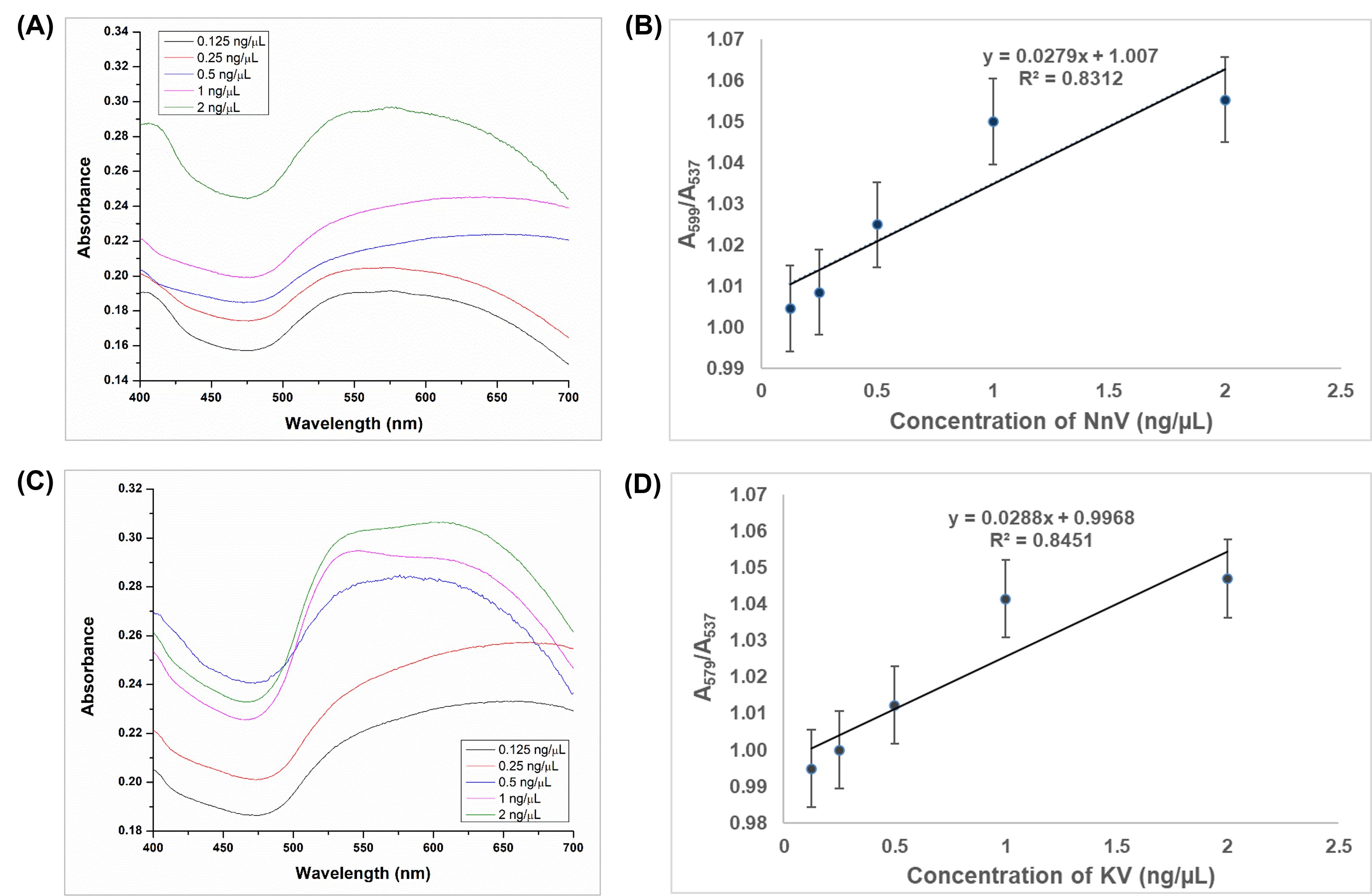

Supplement: S11 Fig — (A) Absorbance spectrum for NnV spiked rat plasma detection by AuNP-FPAb conjugate. Absorbance curves correspond to plasma samples containing 0.125-2 ng/μL NnV; (B) Calibration curve for NnV spiked rat plasma detection at concentrations 0.125-2 ng/μL; (C) Absorbance spectrum for KV spiked rat plasma detection by AuNP-FPAb conjugate. Absorbance curves correspond to plasma samples containing 0.125-2 ng/μL KV; (D) Calibration curve for KV spiked rat plasma detection at concentrations 0.125-2 ng/μL; (E) Absorbance spectrum for NkV spiked rat plasma detection by AuNP-FPAb conjugate. Absorbance curves correspond to plasma samples containing 0.25-4 ng/μL NkV; (F) Calibration curve for NkV spiked rat plasma detection at concentrations 0.25-4 ng/μL; (G) Absorbance spectrum for RvV spiked rat plasma detection by AuNP-FPAb conjugate. Absorbance curves correspond to plasma samples containing 0.125-2 ng/μL RvV; (H) Calibration curve for RvV spiked rat plasma detection at concentrations 0.125-2 ng/μL; (I) Absorbance spectrum for EcV spiked rat plasma detection by AuNP-FPAb conjugate. Absorbance curves correspond to plasma samples containing 0.25-4 ng/μL EcV; (J) Calibration curve for EcV spiked rat plasma detection at concentrations 0.25-4 ng/μL; Error bars indicate mean ± S.D. (n = 3). (TIFF) [file pntd.0012913.s012.tiff]
